# Effects of Environmental Heat Load on Endocannabinoid System Components in Adipose Tissue of High Yielding Dairy Cows

**DOI:** 10.3390/ani12060795

**Published:** 2022-03-21

**Authors:** Gitit Kra, Jayasimha Rayalu Daddam, Uzi Moallem, Hadar Kamer, Majdoleen Ahmad, Alina Nemirovski, G. Andres Contreras, Joseph Tam, Maya Zachut

**Affiliations:** 1 Department of Ruminant Science, Institute of Animal Sciences, Agriculture Research Organization, Volcani Institute, Rishon Lezion 7505101, Israel; gititk@volcani.agri.gov.il (G.K.); jayasimha@volcani.agri.gov.il (J.R.D.); uzim@volcani.agri.gov.il (U.M.); kamer.hadar@gmail.com (H.K.); 2 Department of Animal Science, The Robert H. Smith Faculty of Agriculture, Food and Environment, The Hebrew University of Jerusalem, Rehovot 76100, Israel; 3 Obesity and Metabolism Laboratory, Institute for Drug Research, School of Pharmacy, Faculty of Medicine, The Hebrew University of Jerusalem, Jerusalem 9112001, Israel; majdolee.ahmad@mail.huji.ac.il (M.A.); alina.nemirovskai@mail.huji.ac.il (A.N.); yossi.tam@mail.huji.ac.il (J.T.); 4 Department of Large Animal Clinical Sciences, Michigan State University, East Lansing, MI 48824, USA; contre28@msu.edu

**Keywords:** heat stress, endocannabinoids, CB1, 2-AG, AEA, TRPV1, PPAR-α, adipose tissue, dairy cows

## Abstract

**Simple Summary:**

We hypothesized that environmental heat load (HL) may affect the endocannabinoid system (ECS), a central regulator of metabolism and the stress response, in adipose tissue (AT), plasma and milk of dairy cows. In AT of summer vs. winter calving cows, gene expression of ECS components was decreased, but this was not translated to differences in protein abundance or in levels of endocannabinoids. In late-lactation cows that were not cooled vs. cooled, AT protein abundance of the heat sensitive, and ECS receptor, transient-receptor-potential-cation-channel-subfamily-V-member-1 (*TRPV1*) tended to be lower, and milk levels of 2-arachidonoylglycerol (2-AG) tended to increase in cows that were not cooled; but other ECS components were not different between groups. This suggests that HL is associated with limited alterations in the ECS of AT in dairy cows, either directly or via reduced feed intake.

**Abstract:**

Environmental heat load (HL) adversely affects the performance of dairy cows. The endocannabinoid system (ECS) regulates metabolism and the stress response, thus we hypothesized that HL may affect the ECS of dairy cows. Our objective was to determine the levels of endocannabinoids (eCBs) and gene and protein expressions of the ECS components in adipose tissue (AT) and plasma of early postpartum (PP) and late-lactation cows. In addition, we examined eCBs in milk, and studied the interaction of eCBs with bovine cannabinoids receptors CB1 and CB2. In the first experiment, plasma and AT were sampled from cows calving during summer (S, *n* = 9) or winter (W, *n* = 9). Dry matter intake (DMI) and energy balance (EB) were lower in S vs. W, and relative gene expressions of transient-receptor-potential-cation-channel-subfamily-V-member-1 (*TRPV1*), the cannabinoid receptors *CNR1* (CB1) and *CNR2* (CB2), and monoglyceride lipase (*MGLL*) were decreased in AT of S compared to W. Protein abundance of peroxisome proliferator-activated-receptor-alpha (PPAR-α) was decreased, while tumor-necrosis factor-α (TNF-α) was increased in AT of S vs. W. Other components of the ECS were not different between S and W calving cows. To study whether the degree of HL may affect the ECS, we performed a second experiment with 24 late-lactation cows that were either cooled (CL) or not cooled (heat-stressed; HS) during summer. DMI was lower in HS vs. CL, AT protein abundance of PPAR-α was lower, and TRPV1 tended to be lower in HS vs. CL, but other components of the ECS were not different between groups. Milk levels of 2-arachidonoylglycerol (2-AG) tended to increase in HS vs. CL. Additionally, modeling of the bovine cannabinoid receptors demonstrated their binding to anandamide and 2-AG. Environmental HL, possibly via lower intake, is associated with limited alterations in ECS components in AT of dairy cows.

## 1. Introduction

Environmental heat load (HL), a combination of high ambient temperature and humidity, negatively affects livestock production. In the next decades, climate change is predicted to increase the prevalence and intensity of HL [1]. As a consequence, livestock will be increasingly exposed to heat stress, negatively impacting wellbeing and the sustainability of the animal agriculture industry [2]. Due to their increased metabolic rate, high-yielding dairy cows are extremely sensitive to hot environment [3]. In these animals, chronic HL has detrimental effects on feed intake, milk production and reproductive performance [4]. The transition period from late pregnancy to onset of lactation is characterized by changes in the immune and metabolic functions. Around parturition, HL contributes to systemic inflammation by increasing the expression of pro-inflammatory cytokines in blood cells [5,6], leading to immune dysfunction [7]. Key modulators of the metabolic function such as adipose tissue (AT) are also affected by HL; during seasonal HL, subcutaneous AT of periparturient cows exhibits enrichment of proteins related to the oxidative stress response pathway such as Nrf2 [8].

The endocannabinoid system (ECS) plays a crucial role in activation of key processes for AT metabolic function including lipogenesis and adipogenesis as well as inhibition of lipolytic activity [9]. The ECS is a central regulator of whole body metabolism and energy homeostasis by reducing the duration and intensity of negative energy balance in mammals [10]. However, its role in ruminant physiology is only recently emerging [9,11,12]. The cannabinoid-1 (CB1) receptor is abundant in the brain and in peripheral tissues of rodents and humans, and can be present on adipocytes as well as on infiltrating immune cells in AT [13,14,15]. The enzymes that synthesize and degrade the ECS ligands, the endocannabinoids (eCBs), were also detected in AT [15,16]. The activation of CB1 receptor in adipocytes triggers de novo biosynthesis of fatty acids, accumulation of triglycerides, and minimizes lipolysis [10]. We have demonstrated that dairy cows calving in summer that exhibited a high degree of AT lipolysis postpartum (PP) had 2-fold elevated levels of the main eCBs anandamide (AEA) and 2-arachidonoylglycerol (2-AG) compared to prepartum levels. In the same group of cows, the endogenous precursor and breakdown product arachidonic acid (AA) was also elevated in AT collected PP compared to precalving samples [11]. These findings suggested a correlation between the ECS and the metabolic response of AT in PP dairy cows.

The ECS is also a potent modulator of inflammatory responses and immune function in mammals. For example, AEA and 2-AG have both anti- and pro-inflammatory functions [17,18,19]. The cannabinoid-2 (CB2) receptor, expressed by various cells of the immune system, can also modulate the release of cytokines and appears to have a critical role in immune regulation and function [20]. In fact, CB2 has been found to play a complex role in regulating immune cell migration, proliferation, and numerous effector functions [21]. Recently, it was demonstrated that the expression of pro-inflammatory genes (TNF-α, IL-6, IL-1β) was higher in AT of cows that experienced a high degree of AT lipolysis at 21 and 42 d PP, and this was coupled with higher expression levels of CB2 receptor, as well as N-acyl phosphatidylethanolamine-specific phospholipase D (NAPEPLD) and fatty acid amide hydrolase (FAAH), the enzymes that are responsible for AEA synthesis and degradation, respectively [22]. The mechanisms for ECS’s anti-inflammatory effects in bovines are unclear. However, a prominent target is the nuclear receptor PPAR-α that has an anti-inflammatory activity and at the same time modulates AT metabolism. [23]. PPAR-α is robustly activated by the eCB-like molecules, palmitoylethanolamide (PEA) and oleoylethanolamide (OEA) [23]. Another receptor related to the ECS is the heat-sensitive transient-receptor-potential-cation channel-subfamily-V-member-1 (TRPV1), which is a ligand-gated ion channel that plays a key role in modulation of the sensation of pain and thermal hyperalgesia [24]. TRPV1 has been shown to be expressed in the AT [25], and both thermal heat [25] and AEA [26,27], among other activators, activate the TRPV1 receptor.

We hypothesized that HL during summer may affect the ECS in dairy cows, possibly via TRPV1, and we aimed to determine the levels of eCBs and gene and protein expressions of the ECS components in AT of early PP and late-lactation cows. In addition, we studied the effect of the degree of HL on TRPV1 and ECS in AT of dairy cows during summer as well as the interaction of 2-AG and AEA with bovine CB1 and CB2 receptors.

## 2. Materials and Methods

### 2.1. Animals and Experimental Procedures and Collection of Blood and Milk

#### 2.1.1. Seasonal Effects on eCB ‘Tone’ in Adipose Tissue of Early Postpartum Cows (S vs. W)

The experimental protocol was performed in accordance with the approval of the Volcani Center Animal Care Committee (approval numbers IL 697/17 and IL 318/19). Eighteen high-yielding, 255 ± 5 d pregnant dry multiparous Israeli-Holstein dairy cows with an average parity of 3.5 (range 2–7) that were available at the Volcani Dairy farm (Rishon LeZion, Israel) during the summer season (*n* = 9, August–September) or winter season (*n* = 9, January–February) participated in this experiment. The cows calving in summer were exposed to five cooling sessions per day postpartum, and were part of a study described in Zachut et al. (2020) [28]. Relative humidity (RH) and ambient temperature were recorded every 3 h by the Israel Meteorological Service (Bet Dagan, Israel), and temperature humidity index (THI) was calculated as described [29]. The average calculated THI was 77.5 in S and 57.0 in W. All cows were housed in a covered loose pen with an adjacent outside yard, and were fed ad libitum once a day at 1100 h with a standard Israeli lactating cows’ diet containing 16.5 percent crude protein and 1.78 Mcal NE_L_ per kg dry matter. The cows were group-housed from calving to 21 d PP in a barn that was equipped with a real-time electronic individual feeding system. Each feeding station included an individual identification system that allowed each cow to enter a specific feeding station and automatically recorded each meal (ID tag; S.A.E. Afikim, Kibbutz Afikim, Israel). Body weight (BW) and milk production were recorded three times daily (at 05:00, 13:00, and 20:00 h) at the milking parlor (SAE, Kibbutz Afikim, Israel). In addition, milk samples were collected every two weeks and analyzed for milk fat, protein, lactose, and urea by infrared analysis (standard IDF 141C:2000) at the laboratories of the Israeli Cattle Breeders’ Association (Caesarea, Israel) [30]. Energy balance was calculated according to NRC (2001) [31]. All cows were observed 5 to 10 d after calving by a veterinarian according to the farm’s routine management and treatments were recorded. During the routine checkup, 4 cows in S were diagnosed by the veterinarian with mild metritis, were treated once, and recovered; one W cow had ketosis, was treated intravenously with a dextrose infusion, rechecked 4 d later and was found healthy. Blood samples were collected twice a week at 07:00 h, and centrifugation was done at 4000× *g* for 15 min for plasma and the separated plasma stored at −80 °C for further analysis. BCS (scale 1–5) was determined at the day of AT biopsy by a single technician [32].

#### 2.1.2. Comparing Not Cooled (Heat-Stressed) vs. Cooled Late-Lactation Cows in Summer (HS vs. CL)

The protocol of this experiment was approved by the Volcani Center Animal Care Committee (approval number IL 318/19). In the second experiment, twenty-four multiparous Israeli-Holstein dairy cows at late-lactation (on average 272 ± 7 days in lactation) with an average parity of 3.5 (range 2–7) were allocated for this experiment at the Volcani dairy farm during the summer season. Cows were divided into two treatments according to milk production, BW and parity into: (1) cooled cows (CL; *n* = 12)—exposed to five cooling sessions per day in the holding area of the milking parlor; and (2) Heat stress (HS; *n* = 12)—were exposed to the ambient weather without cooling sessions or fans during the experiment. For CL cows, each cooling session lasted 45 min, comprising repeated cycles of 45 s of showering and 4 min of forced ventilation without showering. The cooling area, which was part of the holding pen, was 12 × 9 m (108 m^2^) and included 30 sprinklers (720 L/h each), 3 large fans (198.1 cm in diameter; capacity: 120,000 m^3^ of air/h each) located on the back side of the cooling area, and 4 small fans (50.8 cm in diameter; capacity: 8800 m^3^ of air/h each) hung at the top of the cooling area. The experiment was conducted during peak summer (August) for a period of 14 days. Relative humidity (RH) and ambient temperature were recorded for every 3 h by the Israel Meteorological Service (Bet Dagan, Israel), and THI was calculated as described in Bohjmanova et al., 2007 [29]. The average calculated THI during the experiment was 78.2. Respiratory rate (RR) and rectal temperature (RT) were recorded twice weekly. On each day, measurements were taken at 07:00 and 15:00 h. Rectal temperature was recorded by a clinical thermometer inserted 12 cm and was held on the rectum wall for 1 min. Respiration rates were measured by counting flank movements during 1 min, as recorded with a stopwatch. All cows were housed in a group with an adjacent outside yard and fed ad libitum with a typical Israeli diet containing 16.5 percent crude protein and 1.78 Mcal NEL per kg dry matter once a day at 1000 h. BW and milk production (at 05:00, 13:00, and 20:00 h) were recorded as described above. In addition, milk samples from three consecutive milking sessions were collected on day 9 of the trial and evaluated for milk fat, protein, lactose, and urea using infrared analysis (standard IDF 141C:2000) at the Israeli Cattle Breeders’ Association’s laboratory (Caesarea, Israel). Additional milk samples were taken at the noon milking at the day of milk sampling and immediately frozen in −80 °C. Energy balance was calculated according to NRC (2001) [31]. Blood samples were collected at days 1, 6, 8, 10 and 13 of the experiment at 07:00 h, and separated plasma by centrifugation at 4000× *g* for 15 min and then stored at −80 °C for further analysis. BCS (scale 1–5) was determined at the day of AT biopsy by a single technician [32].

### 2.2. Analysis of Circulating NEFA, Oxidative Stress and Inflammatory Markers

Non-esterified fatty acids (NEFA), the oxidative stress marker malondialdehyde (MDA), cortisol, and TNF- were measured in plasma samples from both investigations. Plasma NEFA was measured as described in [8]; the intra-assay CV for NEFA was 6.7 percent. The intra- and inter-assay CV were 9.5 percent and 2.6 percent, respectively, when plasma MDA concentrations were determined using a fluorimetric thiobarbituric acid reactive assay [33]. A Bovine TNF- duoset ELISA kit (R&D Systems, Inc., Minneapolis, MN, USA) was used to detect plasma TNF- concentrations; the intra- and inter-assay CVs were 9.4 and 5.7 percent, respectively. ELISA was used to measure cortisol levels in the blood (EIA1887, DRG International, Inc., Springfield, NJ, USA). In the first experiment, radioimmunoassay was used to detect plasma insulin concentrations (MP Bio-medicals, Solon, OH, USA). The Cobas C111 Autoanalyser was used in the second experiment to determine plasma glucose concentrations (Roche Holding GmbH, Grenzach-Wyhlen, Germany).

### 2.3. Biopsy of Subcutaneous AT

In the first experiment on seasonal effects, subcutaneous AT biopsies from the fat pad around the pin bones were taken from each cow (*n* = 9 from each season) at day 7 ± 2 postpartum, as previously described [34]. At the day of AT biopsy all cows were without clinical signs of disease. In the second experiment of HS vs. CL, the AT biopsies were conducted at day 13 or 14 of the experiment, from a subset of 6 CL and 6 HS cows. In short, the biopsy site was prepped by clipping, cleaning, and sterilizing a 5-cm patch of skin on one side of the pin bone. Cows were sedated with 1 mL of 2 percent Sedaxylan (xylazine base, 20 mg/mL; Eurovet Animal Health, AE Bladel, The Netherlands) administered intramuscularly. An 8-mL subcutaneous injection of 2 percent lidocaine HCl (Esracain 2 percent, 200 mg per 10 mL; Rafa Laboratories Ltd., Jerusalem, Israel) was used to anesthetize the biopsy site, and aseptic procedures were used to make a 1.5- to 2.5-cm blade incision through the skin and subcutaneous tissues. Tweezers were used to catch four samples of nearly 40 mg of fat tissue, which were then cut off using scissors and rinsed in saline before being snap frozen in liquid nitrogen and stored at −80 °C.

### 2.4. Measurements of eCBs in AT

In both experiments, eCBs levels were measured in all AT samples and in plasma at the day of AT biopsy (*n* = 9 per group in S vs. W, and *n* = 6 per group in CL vs. HS). In the second experiment, in order to assess whether the degree of HL affects milk eCBs, we analyzed in a subset of milk samples from 10 CL and 10 HS cows. Levels of 2-AG, AEA, PEA, OEA, and AA in AT, plasma and milk were isolated, purified, and measured by the stable isotope dilution LC-MS/MS method as described in [11,35]. The values are shown in fmol/mg tissue (wet weight), pmol/mg tissue (wet weight), or nmol/mg tissue (wet weight), and for the plasma or milk values are shown in nmol/mL, pmol/mL (picomole/mL), and fmol/mL (femtomole/mL).

### 2.5. Relative Gene Expression in AT by Quantitative Real-Time PCR

For RNA extraction, 40 mg of AT samples (*n* = 9 per group in S vs. W, and *n* = 6 per group in CL vs. HS) were homogenized in 1 mL of lysis solution with one metal bead using the RNeasy lipid tissue mini kit (Qiagen, Hilden, Germany). The 260/280 ratio of the RNA quality was greater than 1.85. A cDNA reverse transcription kit was used to generate first-strand cDNA (Applied Biosystems, Foster City, CA, USA). Real-time PCR was used to detect specific mRNA transcripts analyzed in duplicates using a StepOnePlus instrument (Applied Biosystems) and the SYBR green PCR mix (Invitrogen, Carlsbad, CA, USA). We looked at the expression of the following eCB-related genes: cannabinoid-1 receptor (*CNR1*), 2 (*CNR2*), *MGLL*, fatty acid amide hydrolase (*FAAH*), and N-Acylphosphatidylethanolamine phospholipase D (*NAPEPLD*). These primers were according to Zachut et al. (2018) [11]. In addition, we examined the expression of inflammatory genes: *TNFA*, cluster of differentiation 68 (*CD68*), nuclear factor kappa-light-chain-enhancer of activated B cells (*NFKB*), peroxisome proliferator-activated receptor alpha (*PPARA*) and transient receptor potential cation channel subfamily V member 1 (*TRPV1*), as well as genes related to the NrF2-oxidative stress response: mitogen-activated protein kinase (*MAP2K*), superoxide dismutase-1 (*SOD1*), NrF2, stress induced phosphoprotein-1 (*STIP1*) and glutathione S-transferase Mu 1 (*GSTM1*) [8,28]. Data of AT samples were standardized for the presence of two reference genes, *GAPDH* and *HPRT1*, according to MIQE recommendations, and primers were confirmed before use [36].

### 2.6. Protein Abundance in AT by Immunoblot Analysis

Protein abundance was determined in AT samples (*n* = 9 per group in S vs. W, and *n* = 6 per group in CL vs. HS) by immune blotting method. The protein concentrations of AT were measured by Bradford assay (Bio-Rad protein quantification kit). Subsequently, under reducing conditions, SDS-PAGE was used to resolve 20 g of sample in Laemmli loading buffer, which was then transferred to nitrocellulose membrane with the following antibodies: MGLL (1:200, ab24701, Abcam, Cambridge, UK), β-actin (1:1000, ab46805, Abcam Biotech, Cambridge, UK), CB1 (1:200, ab23703, Abcam Biotech, Cambridge, UK), FAAH (1 µg/mL, ARP33121_P050, Aviva Systems Biology, San Diego, CA, USA), CB2 (ADI-905-820-100, Enzo, Farmingdale, NY, USA), PPAR-α (ab24509, Abcam Biotech, Cambridge, UK), TRPV1 (1 µg/mL, WH0007442M1, Sigma, St. Louis, MO, USA) and TNF-α (OACA04183, Aviva Systems Biology, San Diego, CA, USA). For protein detection, a 1:10,000 dilution of goat anti-rabbit HRP conjugated secondary antibody (Jackson Immunoresearch 111-035-003, West Grove, PA, USA) was used for chemiluminescence reaction. ImageJ software was used to process and analyze the data (NIH, Bethesda, MD, USA). β-actin was used to equalize the signal bands.

### 2.7. Homology Modeling and Active Site Prediction of CB1 and CB2

To predict CB1 and CB2 receptors structures, the protein sequences were collected from UNIPROT database using *bos taurus* as reference (CB1: Uniprot_KB Accession Id: Q17QM9 and CB2: Uniprot_KB Accession Id: E1B9P2). The domain regions of these proteins were predicted by SBASE server (pongor.itk.ppke.hu) and searched against BLAST (blast.ncbi.nlm.nih.gov) accessed at 11 October 2021 using PDB [37]. The CB1 and CB2 receptor sequences aligned with their templates respectively using ClustalX software and structures were generated by MODELLER9V7 [38]. Among the generated structures, the least energy models of CB1 and CB2 receptors were selected for further studies. Using NAMD and CHARMM27 force field, these predicted models were stabilized in according to Daddam et al. [39]. The reliability of CB1 and CB2 structures were confirmed by Ramachandran plot server and PROCHECK server. ERRAT, structure evaluation server used to check the environmental profile of the structures [40]. The binding sites of CB1 and CB2 receptors were identified by CASTp server (sts.bioe.uic.edu/castp) accessed at 26 October 2021 and also by comparing templates with structure.

#### Molecular Interaction of 2-AG and AEA with CB1 and CB2 Receptors

The interaction of 2-AG and AEA with CB1 and CB2 receptors was done using GOLD 3.0.1 software, through docking studies [41]. 2-AG and AEA compounds were docked to the active site of CB1 and CB2 receptors to study the possibility of interaction mechanism. Following docking, each protein-compound complex’s distinct binding poses were chosen and their binding energies were investigated. The complex’s most energetic conformation was chosen, and docking studies were examined [42]. To find the possible interaction of 2-AG, AEA on bovine CB1 and CB2 receptors, CB1 and CB2 sequences from Bos taurus structures were predicted. CB1 receptor contains RelB antitoxin-like domain (37–91 amino acids) and rhodopsin-like GPCR super family domain (133–397 AA). CB2 receptor contains rhodopsin-like domain (50–229 amino acids). The CB1 receptor structure was developed by using CB1 receptor of synthetic construct structure (PDB ID: 6WSK_A), showed similarity (95.1%) with CB1 from Bos taurus. The CB2 receptor structure was generated by Cryo-EM structure of GPCR (PDB code: 6KPF_R) structure of Homo sapiens, showed similarity (82.7%) with CB2 from Bos taurus.

### 2.8. Statistical Analysis

In the seasonal effects experiment, plasma concentrations of insulin, NEFA, cortisol, MDA and TNF-α, as well as milk production, FCM, DMI and energy balance were analyzed by repeated measurements PROC MIXED, using the following model:

Y_ijkl_ = µ + S_i_ + C(S)_ij_ + DIM_ijk_ + E_ijkl_, (1)

where µ = overall mean; S_i_ = season effect (i = summer or winter); C(S)_ij_ = cow j nested in season i; DIM_ijk_ = day in lactation as a continuous variable; E_ijkl_ = random residual.

In CL vs. HS experiment, continuous variables (plasma measures, milk, DMI and EB) were analyzed by PROC MIXED using a similar model as described above, where S_i_ = CL effect (i = cooled or Heat stress); C(S)_ij_ = cow j nested in treatment i; DIM_ijk_ = day in lactation as a continuous variable; E_ijkl_ = random residual.

Protein abundances and mRNA expressions from each experiment and BCS values at day of AT biopsy were evaluated by SAS GLM (version 9.2, 2002). The eCB levels in plasma, milk and AT are shown as the mean ± SEM, and were analyzed by unpaired two-tailed Student’s *t*-test.

## 3. Results

### 3.1. Heat Load Affects Plasma Indicators of Metabolism, Inflammation, Oxidative Stress, and Lactation Performance in Periparturient Cows (S vs. W)

As shown in Table 1, dry matter intake (DMI) was lower in S than in W during the first month in lactation (*p* < 0.001), milk production was not significantly changed in S compared to W (*p* = 0.11), and fat-corrected milk (FCM, 4%) was lower in S than in W (*p* = 0.02). The average calculated energy balance (EB) was lower in S than in W cows PP (*p* = 0.01; Table 1). The average body condition score (BCS) at 7 d PP was 2.99 in S and 3.04 in W cows (SEM = 0.19; *p* = 0.84). Plasma non-esterified fatty acids (NEFAs) concentrations were not different between S and W calving cows, and plasma insulin concentrations tended to be lower in S (*p* = 0.06; Table 1). No differences in oxidative stress marker malondialdehyde (MDA) or in cortisol concentrations were found between seasons, but plasma TNF-α concentrations were 3.4-fold higher in S than in W (*p* = 0.001; Table 1).

### 3.2. Heat Load Impacts Transcription of ECS Components in AT of S vs. W Calving Cows

In AT biopsies obtained at 7 d PP, the relative mRNA expression levels of *CNR1* and *CNR2* decreased by 55% (*p* = 0.01) and 53% (*p* = 0.009) in S compared to W, respectively (Table 2). The expression of the 2-AG degrading enzyme, *MGLL*, in AT was reduced by 56% in S than in W (*p* = 0.03), while the relative expression levels of *FAAH*, *NAPEPLD* and *PPARA* were not different between seasons (Table 2). The expression of *TRPV1* was 43% lower in S than in W AT (*p* = 0.001), while *TNFA*, *CD68*, and *NFKB* were not different between seasons (Table 2). The relative expression of the Nrf2-oxidative stress response genes *SOD1*, *NRF2*, *STIP1* and *GSTM1* showed no significant difference between seasons; however, the expression of *MAP2K* was higher in S than in W AT (*p* = 0.009; Table 2).

### 3.3. Heat Load and ECS Components’ Protein Abundance in AT of S vs. W Calving Cows

**Figure 1:**
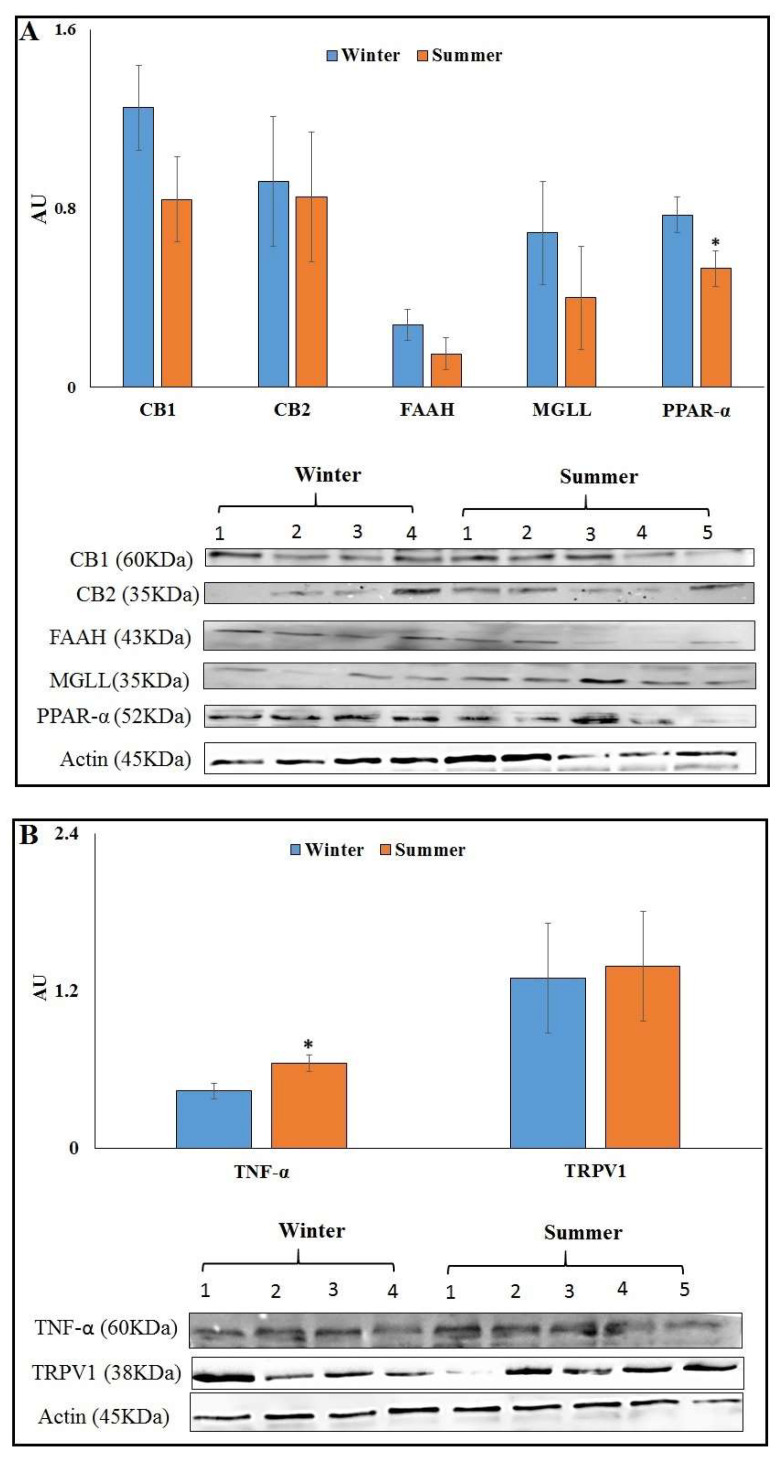
Protein abundance of endocannabinoid system (ECS) components in AT at 7 d PP from cows calving in winter (*n* = 9) or summer (*n* = 9). (**A**) Presented are the average abundances of CB1, CB2, FAAH, MGLL and PPAR-α, and representative immunoblots of 5 S and 4 W AT. (**B**) Presented are the average abundances of TNF-α and TRPV1, and representative immunoblots of 5 S and 4 W AT. * Significant with *p* ≤ 0.05.

The changes measured in the mRNA expression were not translated to differences in the protein abundances of CB1, CB2, FAAH and MGLL in AT, whereas the protein abundance of PPARα decreased by 31.1% in S than in W (*p* = 0.04) (Figure 1A). The abundance of TNFα increased by 44.4% (*p* = 0.04), and TRPV1 was not different in S compared to W AT (Figure 1B). Full blots are provided in Supplementary Figure S1.

### 3.4. Levels of eCBs in AT and Plasma at 7 d PP of Summer vs. Winter

The average levels of the eCBs AEA, 2-AG, OEA, PEA and AA in plasma and AT collected at d 7 PP are shown in Table 3. As shown, no significant differences were observed between seasons in the levels of eCBs in AT or in plasma of S vs. W cows.

### 3.5. Abating Heat Load by Cooling Affects Idices of Heat Stress in Late-Lactation Cows (HS vs. CL)

In the second experiment, DMI (*p* < 0.001), milk production (*p* = 0.02), and FCM 4% (*p* = 0.05) were decreased in HS compared to CL cows (Table 4). The average calculated EB was lower in HS vs. CL cows (*p* < 0.001; Table 4). Plasma NEFAs concentrations tended to be lower in HS vs. CL cows (*p* = 0.10). No changes in plasma glucose, cortisol, TNF-α concentrations and MDA levels were observed in HS vs. CL, and no significant change in BW gain was observed in HS vs. CL late-lactation cows (Table 4). The average BCS was 2.90 and 3.15 for CL and HS, respectively (SEM = 0.22, *p* = 0.45). Rectal temperatures in morning and afternoon were higher in HS than in CL (*p* < 0.001), and respiration rate was higher in morning (*p* = 0.005) and tended to be higher in afternoon (*p* = 0.08) in HS vs. CL (Table 4). The average THI, rectal temperatures and respiration rates in this experiment are within the range of moderate heat stress [43], and the significant differences between groups in feed intake, milk production, rectal temperature and respiration rates indicate that the HS-cows were experiencing a higher degree of heat stress than the CL cows.

### 3.6. Minimizing Heat Load by Cooling Did Not Affect Gene Expression of ECS Components in AT of HS vs. CL

The relative mRNA expression levels of ECS components: *CNR1*, *CNR2*, *MGLL*, *FAAH*, *NAPEPLD* and *PPARA* as well as the expression of inflammatory genes *TRPV1*, *TNFA*, *NFKB*, and the Nrf2-oxidative stress response genes *MAP2K* and *STIP1* were not significantly different in AT of HS vs. CL late-lactation cows (Table 5).

### 3.7. Minimizing Heat Load by Cooling Affects Protein Abundance of ECS Components in AT of HS vs. CL

**Figure 2:**
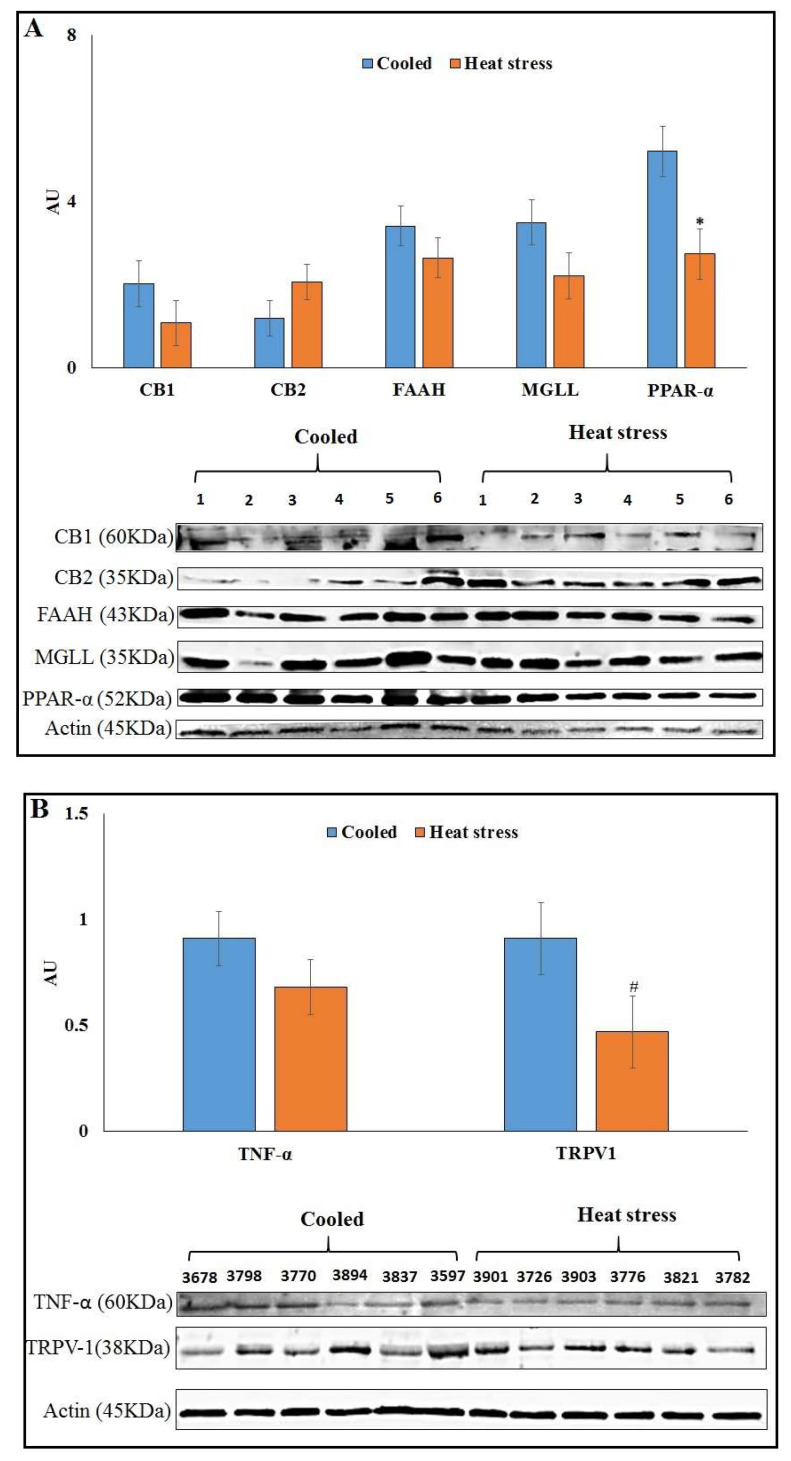
Protein abundance of ECS components in AT of late lactation cooled (*n* = 6) or heat-stressed cows (*n* = 6). (**A**) Presented are the average abundances of CB1, FAAH, MGLL and PPAR-α and immunoblots. (**B**) Protein abundance of TNF-α and TRPV1 in AT of late lactation cooled (*n* = 6) or heat-stressed cows (*n* = 6). * Significant with *p* ≤ 0.05. # Statistical tendency with *p* = 0.1.

Among the protein abundances of ECS components, only PPAR-α showed a significant decrease (*p* = 0.02) in HS compared to CL AT, whereas no significant differences of CB1, FAAH, MGLL (Figure 2A) or CB2 were detected in AT. The abundance of TRPV1 tended to be lower (*p* = 0.10) in AT of HS vs. CL cows, whereas TNF-α showed no difference in AT (Figure 2B).

### 3.8. Levels of eCBs in AT, Plasma and Milk of HS vs. Cooled Cows

The average concentrations of the eCBs AEA, 2-AG, OEA, PEA and AA in milk, plasma and AT are presented in Table 6. As shown, no significant differences of eCBs were observed in plasma and AT between HS vs. CL cows. However, in milk, the levels of 2-AG were 72.2% higher and tended to be increased in HS vs. CL cows (*p* = 0.06; Table 6).

### 3.9. Modeling the Bovine CB1 and CB2 Receptors and Interactions of 2-AG and AEA

**Figure 3:**
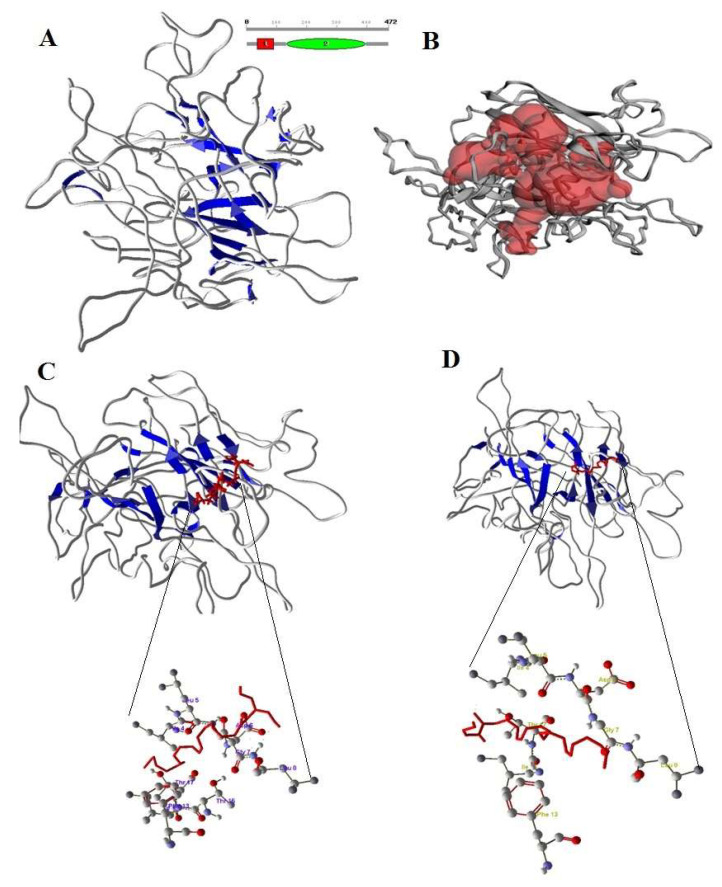
(**A**) Structure of CB1 with sheets (blue color). (**B**) Active site of CB1 in red color pocket in the structure. (**C**) Docking of 2-AG (red color) and Amino acids of CB1 involved in docking with 2-AG. (**D**) Docking of AEA (red color) with CB1 and Amino acids of CB1 involved in docking with AEA.

**Figure 4:**
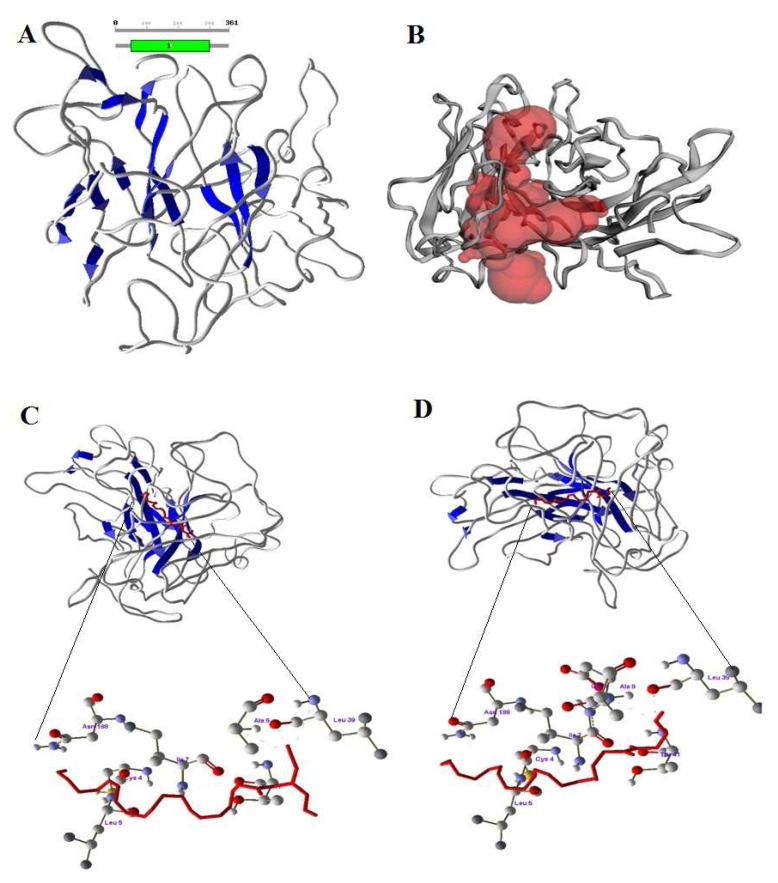
(**A**) Structure of CB2 with sheets (blue color) (**B**) Active site of CB2 in red color pocket in the structure (**C**) Docking of 2-AG (red color) and Amino acids of CB2 involved in docking with 2-AG. (**D**) Docking of AEA (red color) with CB2 and Amino acids of CB2 involved in docking with AEA.

The predicted CB1 and CB2 structures, after stabilizing by molecular dynamics, validated and confirmed the reliability by Ramachandranplot server using PROCHECK server (Figure 3A and Figure 4A). The predicted binding sites of CB1 and CB2 receptors are shown in Figure 3B and Figure 4B. The designed 2-AG and AEA showed good interaction with CB1 and CB2 at the binding region. The docked conformations of 2-AG and AEA with the CB1 receptor binding site are shown in Figure 3C,D. The amino acids LYS2 and ILE16 formed a strong hydrogen bond with 2-AG and AEA. In the bonding of AEA, two hydrogen bonds were observed between hydrogen atom of LYS2 with O1 and O2 of AEA, whereas one hydrogen bond was observed between hydrogen atom of ILE16 and O4 of 2-AG.

The CB2 amino acids ILE7, ALA9 and LEU39 formed a strong hydrogen bonding with 2-AG and AEA (Figure 4C,D). In the bonding of AEA, three hydrogen bonds were observed between the oxygen atom of ILE7 with H51, hydrogen atom of ALA9 with O2 and oxygen atom of LEU39 with H62 of AEA, whereas three hydrogen bonds were observed between the hydrogen atom of THR41 with O2, oxygen atom of LEU39 with H22 and hydrogen atom of ALA9 with O2 of 2-AG. Based on molecular docking studies it was confirmed that CB1 and CB2 receptors of *bos taurus* have strong interaction with 2-AG and AEA at the binding site, forming a receptor-compound complex with amino acids of receptors.

## 4. Discussion

### 4.1. Effects of HL on Feed Intake, Metabolic Response, Oxidative Stress and Inflammation and the Possible Role of the ECS in These Responses

In the first experiment, cows calving during S HL had lower feed intake during the first month PP compared to W cows, which could explain their negative EB; this effect of lower intake and lower EB was also evident in the second experiment with HS vs. CL late-lactation cows. Reduced feed intake during HL is well described in previous studies [44]. One limitation of our study is that we cannot determine whether the changes we observed in ECS components are a direct effect of the HL or related to the reduced intake and the associated metabolic adaptations during HL. Thus, to fully understand this relationship, additional studies using other experimental models such as pair feeding are required. Nonetheless, our findings highlight the possible link between environmental factors and ECS activity in dairy cows.

We found lower gene expression of *CNR1* in AT of summer-calving cows that had lower feed intake, but not in AT of HS late-lactation cows that also had lower intake com-pared to CL. CB1 antagonists that are restricted to the periphery (not crossing the blood-brain barrier) have been shown to reduce food intake and body weight in rodents [45,46]. Experiments in heat-stressed chickens indicate that lower hepatic eCB levels are associated with lower feed intake, as eCBs reaching the brain would stimulate feeding [47]. In rodents, adipocyte-specific inducible genetic deletion of CB1 receptor has been shown to protect mice from diet-induced obesity and associated metabolic alterations and to reverse the phenotype in already obese mice [48]. Here, we observed lower feed intake in the summer-calving cows and lower *CNR1* expression in AT without differences in plasma or AT concentrations of eCBs, and no differences in *CNR1* expression in AT of late-lactation HS cows. These results could be possibly explained by the single timing of blood and AT sampling that might have been insufficient to find changes in their levels due to the large variance between cows, or it could suggest that in early lactation cows circulating eCBs and AT levels are not a major effector on feed intake. Together, the few changes that we found in ECS components in HS cows may indicate that the effects of HL on the ECS in AT are limited in their importance; however, more research is required to elucidate this point.

The lack of difference in plasma NEFAs or MDA concentrations in both experiments may indicate that the degree of AT lipolysis was similar among seasons and in CL vs. HS cows. The tendency for lower plasma insulin concentrations in S cows compared to W cows can be attributed to the lower feed intake during summer. Plasma cortisol concentrations were similar between seasons and in HS vs. CL cows, which is in contrast to our previous findings of increased plasma cortisol during heat load [8]. NAD(P)H dehydro-genase [quinone] 1 (NQO1), a critical oxidative stress-response gene regulated by Nrf2, was shown to be higher in S AT than in W AT [8], which could explain our finding of higher *MAP2K* expression in S AT. Together, it might be suggested that the minor changes in oxidative stress may be related to the lack of increase in plasma cortisol in the present experiment. Heat stress has been implicated in increasing inflammation in dairy cows [5,6], and indeed the cows calving during S had increased plasma TNF-α concentrations compared to W-calving cows. In the AT, the protein abundance of pro-inflammatory TNF-α was increased and the abundance of the anti-inflammatory PPAR-α was decreased in S vs. W; however, at the mRNA level we did not see increased expression of the pro-inflammatory genes in S AT (*TNFA*, *CD68, NFKB*). The changes at the protein level could suggest that HL may increase AT local inflammation. It is important to note that the reduction in gene expressions of *CNR1*, *MGLL* and *TRPV1* may be part of the physiological response to lower the degree of inflammation in AT of early PP cows; however, more research is required on this subject. In contrast, in late-lactation cows, no significant change of *TNFα* gene expression or protein abundance was observed in HS vs. CL cows, which could be related to the positive EB and BW gain that occurred in the second experiment. Recently, a higher expression of TNF-α, IL-6 and IL-1β, coupled with increased expression of CB2 and NAPEPLD and lower expression of FAAH, was reported in AT of cows that exhibited increased lipolysis compared to cows with a lesser degree of AT lipolysis at 21 and 42 days postpartum [22]. This was in agreement with our previous findings of a tendency toward a higher expression of CB1 and CB2 in AT of cows with a high degree of BW loss PP that also had increased plasma TNF-α concentrations [11]. Collectively, we suggest that further studies should examine the association between heat stress, inflammation and the ECS in dairy cows.

### 4.2. Effects of HS on the ECS Receptors, MGLL and PPAR-α in AT

As mentioned above, in the present study we demonstrated that seasonal HL (either directly or via reduced feed intake) is associated with reductions in the gene expression of the CB1 (*CNR1*) and CB2 (*CNR2*) receptors in AT of S compared to W calving cows. Although not significant, a similar trend of decreased CB1 abundance was observed at the protein level in AT of S vs. W calving cows, possibly suggesting lower abundance of CB1 receptors on adipocytes during HS. There is scarce information on the relationship be-tween the ECS and HL; Joyeux et al. (2002) [49] suggested an involvement of eCBs, acting through CB receptors, and nitric oxide in the cardio protection conferred by heat stress against myocardial ischemia. In a comprehensive review on the neurobiological interactions between stress and the ECS, Morena et al. (2016) [50] concluded that exposure to chronic stress causes a downregulation or loss of CB1 receptors in the brain, which together with our findings of lower CB1 expression in AT of S cows could suggest that chronic HL (weeks to months) may elicit a similar reduction in CB1 receptor in AT of PP cows. It could be that CB1 overactivation following chronic stressors such as heat leads to its downregulation since it is a GPCR receptor, and its internalization/downregulation in these conditions has been reported [51]. The decrease in CB1 receptor expression during chronic stress could be related to the hypothesis of allostatic load [52].

We found reduced gene expression of *MGLL*, the 2-AG degrading enzyme, in AT of S vs. W calving cows. It was previously reported that *MGLL* gene expression decreased in AT of PP cows experiencing higher inflammation and lipolysis [11], which is in accordance with reduced *MGLL* expression in cows suffering from endometritis [53]. A decrease in *MGLL* expression has been suggested as a possible factor leading to improved 2-AG signaling capacity after repeated stress [50,54]. However, the similar protein abundance of the ECS enzymes (MGLL, FAAH, NAPEPLD) we found in AT could suggest that the activity of these enzymes, rather than their abundance, may have been affected by HL, and this issue warrants further investigation. 

The protein abundance of PPAR-α was lower in AT during S compared to W and HS vs. CL cows. PPAR-α is a nuclear receptor with an anti-inflammatory activity that controls metabolism and its dysfunctions [23], and is activated by fatty acid derivatives, which upregulate the catabolic enzyme expression of fatty acid β-oxidation and microsomal ω-oxidation that regulates the transcription of genes necessary for redox balance in oxidative catabolism of fatty acids [23]. The eCB-like molecules, PEA and OEA, are among the most potent endogenous ligands for PPAR-α, and it was speculated that reduction in OEA levels in AT with unchanged PPAR-α may account for lipid accumulation in AT [23]. We found lower abundance of PPAR-α in AT of HS cows vs. CL cows; however, we did not observe changes in OEA and PEA levels in their AT. Therefore, we cannot link the two molecules with the reduction of PPAR-α. In addition, no significant difference among the eCBs, oxidative stress and inflammatory genes was found in our experiment in HS vs. CL cows.

### 4.3. eCB Levels in Plasma, Milk and AT of PP Dairy Cows

In the present experiment we also examined the concentrations of circulating eCBs in plasma, milk and AT of dairy cows. Though no differences were detected among eCB levels, these data are among the first to identify eCBs in milk, plasma and AT of dairy cows that are exposed to a hot and humid environment in summer. Previously, eCB levels in milk from different breeds of dairy cows [55], and in heart and liver tissues from post-mortem 18-month old heifers [56], were reported using similar methods as in the present study. Recently, Kuhla et al. (2020) [12] described the concentrations of plasma eCBs in peripartum cows, showing that plasma 2-AG and AEA levels increased 2-fold in the first month after parturition relative to the prepartum levels. It was suggested that the orexigenic action of these eCBs is to increase metabolic rate and energy intake in order to meet the increased requirements for milk synthesis in early lactation [12]. Kuhla et al. (2020) [12] reported that average plasma AEA concentrations at 7 d PP were ~600 fmol/mL and 2-AG was ~0.003 nmol/mL, which is comparable to our findings. In the present study we did not observe differences in AEA concentrations in AT of HS cows, although the AEA concentrations were double in S compared to W AT. Interestingly, milk 2-AG levels tended to be higher in HS vs. CL cows, and therefore we suggest that levels of eCBs in cows’ milk, plasma and AT should be examined in additional HL studies and in multiple time points.

### 4.4. Effects of HL on TRPV1 in AT

In the present study, decreased gene expressions of the heat sensitive receptor *TRPV1* was found in AT of S vs. W calving cows. As mentioned above, TRPV1 is a ligand-gated ion channel that plays a key role in modulation of the sensation of pain and thermal hyperalgesia [24], and is expressed in the AT [25]. Both thermal heat [25] and the eCB AEA [26], among other activators, activate the TRPV1 receptor. Recently it was shown that AEA activates TRPV1 via a unique lipid pathway at a peripheral binding site [57,58]. In addition, AT B-lymphocytes that regulate the local inflammatory response produce leukotriene B4, which is also a TRPV1 agonist [25]. In our study, protein abundance of TRPV1 tended to be decreased in AT of HS vs. CL late-lactation cows whereas no significant change was observed in early lactation S vs. W cows. Based on our findings we suggest that further studies should examine the possible role of TRPV1 in AT of HS dairy cows.

### 4.5. Molecular Interaction of Bovine CB1, CB2 Receptors with eCBs

To study the interactions of 2-AG and AEA with bovine CB1 and CB2 receptors, we used the in silico method of modeling and docking; our findings show new binding residues of bovine CB1 and CB2 receptors with possible interactions of endocannabinoids. Modeling of protein structures and validating for interaction studies is a common method used previously [59,60,61] and has reliable predictions [62,63,64,65]. Our modeling demonstrates that the important amino acids in the active site formed a large pocket where 2-AG and AEA can bind to bovine CB1 and CB2 receptors. The interaction of CB1 with eCBs was also examined by Saleh et al. [66], who demonstrated the role of active residues in the allosteric mechanism of the CB1 PAM ZCZ011. The CB1 and CB2 interactions with 2-AG and AEA in the present study provide information about the activation of receptors, but more studies are required on the function of eCBs once they bind to their receptors in dairy cows.

## 5. Conclusions

The results from the present study suggest that environmental HL, either directly or via reduced feed intake, has a limited effect on ECS components in the AT of dairy cows, and the heat-sensitive receptor TRPV1 could be related to the association between HL and the ECS in AT. One limitation of this study is the relatively small sample of cows; thus, to fully elucidate the effects of HL on the ECS in dairy cows, more research with a large number of cows is required.

## Figures and Tables

**Table 1:** Dry matter intake, milk production, and energy balance during the first month postpartum (PP), and plasma concentrations of metabolic and stress indicators during the peripartum period in cows calving during winter or summer.

	Winter	Summer	SEM	*p*
Dry matter intake 30 d, kg/d	25.0 ^a^	18.5 ^b^	0.8	<0.0001
Milk production 30 d, kg/d	41.7	37.5	1.7	0.11
Fat corrected milk (4%) 30 d, kg/d	40.7 ^a^	34.0 ^b^	1.9	0.02
Energy balance 30 d, Mcal/d	0.6 ^a^	−4.5 ^b^	1.7	0.01
NEFAs, µEq/L	414.0	437.0	47.7	0.74
Insulin, pg/mL	20.1	14.2	2.0	0.06
Cortisol, ng/mL	7.8	6.8	1.8	0.69
MDA, µM	224.3	388.2	91.6	0.25
TNF-α, pg/mL	375.8 ^b^	1289.3 ^a^	161.6	0.001

NEFAs = Non-esterified fatty acids; MDA = Malondialdehyde; TNF-α = Tumor necrosis factor alpha. ^a,b^ Different letters within a row are significant at *p* ≤ 0.05.

**Table 2:** Relative mRNA expression in adipose tissue (AT) at 7 d PP in cows calving during summer or winter.

RQ	Summer	Winter	SEM	*p*
**ECS genes**				
*CNR1*	0.035 ^a^	0.064 ^b^	0.007	0.01
*CNR2*	0.176 ^a^	0.331 ^b^	0.037	0.009
*MGLL*	0.551 ^a^	0.992 ^b^	0.127	0.03
*FAAH*	0.008	0.010	0.002	0.57
*NAPEPLD*	0.012	0.015	0.003	0.42
*PPARA*	0.073	0.056	0.013	0.36
**Inflammatory genes**				
*TRPV1*	0.210 ^a^	0.487 ^b^	0.051	0.001
*TNFA*	0.003	0.003	0.001	1.00
*CD68*	0.241	0.465	0.093	0.10
*NFKB*	0.148	0.088	0.034	0.24
**Oxidative stress genes**				
*SOD1*	0.519	0.492	0.068	0.78
*NRF2*	0.244	0.151	0.069	0.36
*STIP1*	0.741	0.919	0.088	0.17
*MAP2K*	2.501 ^a^	1.788 ^b^	0.171	0.009
*GSTM1*	0.106	0.086	0.040	0.73

*CNR1* = cannabinoid-1 (CB1) receptor; *CNR2* = cannaboid-2 (CB2) receptor; *FAAH* = Fatty acid amide hydrolase; *MGLL* = Monoglyceride Lipase; *NAPEPLD* = N-acyl phosphatidylethanolamine-specific phospholipase D; *PPARA* = Peroxisome proliferator-activated receptor alpha; *TRPV1* = Transient receptor potential cation channel subfamily V member 1; *TNFA* = tumor necrosis factor alpha; *CD68* = Cluster of Differentiation 68; *NFKB* = Nuclear factor kappa-light-chain-enhancer of activated B cells; *SOD1* = Superoxide Dismutase1; *STIP1* = Stress Induced Phosphoprotein1; *MAP2K* = Mitogen-activated protein kinase; *GSTM1* = Glutathione S-Transferase Mu 1. ^a,b^ Different letters within a row are significant at *p* ≤ 0.05.

**Table 3:** Average concentrations of endocannabinoids (eCBs) in AT and plasma at 7 d PP from cows calving in winter (*n* = 9) or summer (*n* = 9).

	Winter	Summer	SEM	*p*
**Adipose tissue**				
AEA, fmol/mg	1.6	3.3	1.5	0.429
2-AG, nmol/mg	199.7	199.0	49.0	0.992
OEA, pmol/mg	148.2	238.6	84.5	0.461
PEA, pmol/mg	34.3	66.7	21.2	0.295
AA, nmol/mg	1097.0	1023.2	176.7	0.773
**Plasma**				
AEA, fmol/mL	308.6	443.9	67.9	0.178
2-AG, nmol/mL	16.8	13.9	2.1	0.350
OEA, pmol/mL	65.4	62.4	5.4	0.694
PEA, pmol/mL	1698.9	1186.1	579.9	0.544
AA, nmol/mL	251.5	260.8	28.2	0.820

AEA = Anandamide; 2-AG = 2-Arachidonoylglycerol; OEA = Oleoylethanolamide; PEA = Palmitoylethanolamide; AA = Arachidonic acid; fmol = femtomole; pmol = picomole.

**Table 4:** Dry matter intake, milk production, energy balance, plasma concentrations of metabolic and stress indicators, rectal temperature, resting time, respiration rate and rumination time in cooled vs. heat-stressed late-lactation cows.

	Cooled	Heat Stressed	SEM	*p*
Dry matter intake, kg/d	28.7 ^a^	24.8 ^b^	0.6	0.0005
Milk production, kg/d	32.3 ^a^	28.9 ^b^	0.9	0.02
Fat corrected milk (4%), kg/d	28.1 ^a^	24.0 ^b^	1.4	0.05
Energy balance, Mcal/d	15.2 ^a^	12.6 ^b^	0.9	<0.0001
BW gain, kg	3.5	13.2	5.9	0.26
NEFAs, µEq/L	136.0	120.6	6.4	0.10
Glucose, mg/dL	62.8	59.3	1.8	0.19
Cortisol, ng/mL	1.09	1.03	0.04	0.35
MDA, µM	3.8	4.9	0.8	0.38
TNF-α, pg/mL	163.6	154.0	8.4	0.43
Rectal temperature, morning	37.8 ^b^	38.5 ^a^	0.08	<0.0001
Rectal temperature, afternoon	38.4 ^b^	39.1 ^a^	0.08	<0.0001
Resting time, min/d	586.7	543.2	18.7	0.11
Respiration rate, morning	28.6 ^b^	43.1 ^a^	3.2	0.005
Respiration rate, afternoon	48.6	58.6	3.8	0.08
Rumination time, min/d	517.5	512.9	9.8	0.7

NEFAs = Non-esterified fatty acids; MDA = Malondialdehyde; TNF-α = Tumor necrosis factor alpha; BW = Body weight. ^a,b^ Different letters within a row are significant at *p* < 0.05.

**Table 5:** Relative mRNA expression in AT of cooled vs. heat-stressed late-lactation cows.

RQ	Cooled	Heat Stressed	SEM	*p*
**ECS genes**				
*CNR1*	0.033	0.030	0.007	0.80
*CNR2*	0.303	0.283	0.038	0.71
*MGLL*	0.286	0.149	0.091	0.31
*FAAH*	0.007	0.007	0.002	0.94
*NAPEPLD*	0.028	0.017	0.007	0.30
*PPARA*	0.096	0.059	0.027	0.38
**Inflammatory genes**				
*TRPV1*	0.091	0.080	0.015	0.63
*TNFA*	0.004	0.005	0.001	0.73
*NFKB*	0.045	0.038	0.012	0.68
**Oxidative stress genes**				
*STIP1*	0.670	0.691	0.064	0.81
*MAP2K*	1.116	0.746	0.204	0.23

**Table 6:** Average concentrations of eCBs in AT (*n* = 6 per group), plasma (*n* = 6 per group) and milk (*n* = 10 per group) from cooled vs. heat-stressed late-lactation cows.

	Cooled	Heat Stressed	SEM	*p*
**Adipose tissue**				
AEA, fmol/mg	0.3	0.2	0.06	0.27
2-AG, nmol/mg	164.2	159.6	35.6	0.93
OEA, pmol/mg	32.3	25.6	6.5	0.48
PEA, pmol/mg	5.2	6.8	1.9	0.57
AA, nmol/mg	0.5	0.5	0.1	1.00
**Plasma**				
AEA, fmol/mL	95.2	99.8	12.4	0.80
2-AG, nmol/mL	4.7	5.1	0.5	0.57
OEA, pmol/mL	25.4	28.8	6.4	0.71
PEA, pmol/mL	1.3	3.1	1.2	0.31
AA, nmol/mL	178.8	186.0	21.1	0.81
**Milk**				
AEA, fmol/mL	13.4	18.3	5.6	0.55
2-AG, nmol/mL	29.2	50.3	7.5	0.06
OEA, pmol/mL	5.3	5.6	0.8	0.80
PEA, pmol/mL	14.4	11.0	1.8	0.20
AA, nmol/mL	61.6	58.7	8.4	0.81

AEA = Anandamide; 2-AG = 2-Arachidonoylglycerol; OEA = Oleoylethanolamide; PEA = Palmitoylethanolamide; AA = Arachidonic acid.

## Data Availability

Not applicable.

## Supplementary Materials

The following supporting information can be downloaded at: https://www.mdpi.com/article/10.3390/ani12060795/s1, Figure S1: Full blots.
