# Supplementary material for: Effects of Environmental Heat Load on Endocannabinoid System Components in Adipose Tissue of High Yielding Dairy Cows
Source: Animals (Basel). 2022 Mar 21;12(6):795. doi: 10.3390/ani12060795 (PMC8944798; doi:10.3390/ani12060795)

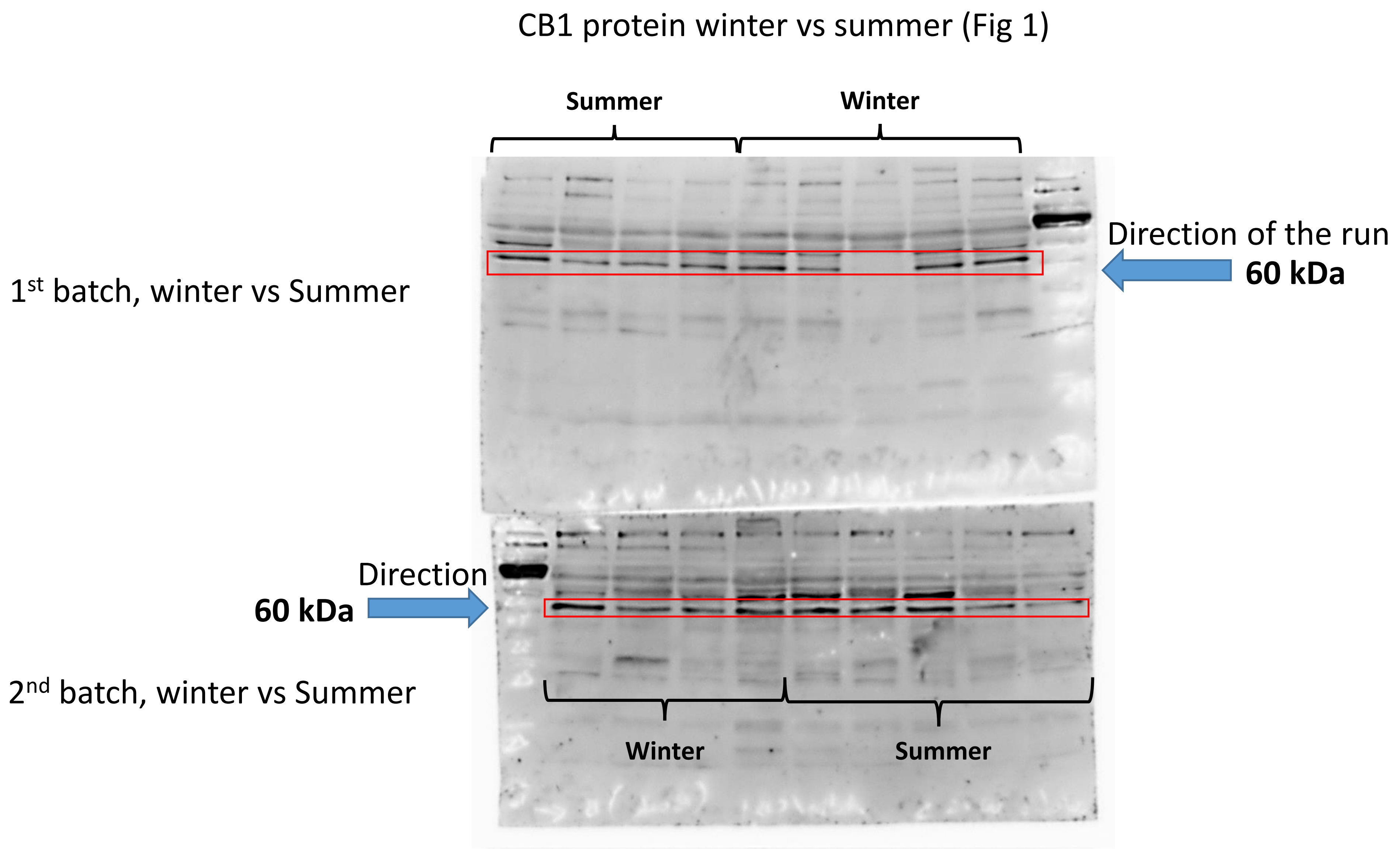
CB1 protein winter vs summer (Fig 1)
Summer
Winter
1st batch, winter vs Summer
Direction of the run
60 kDa
Direction
60 kDa
2nd batch, winter vs Summer
Winter
Summer

CB2 protein Winter vs. Summer (Fig 1)

35 kDa

1st batch, winter vs Summer

35 kDa

2nd batch, winter vs Summer

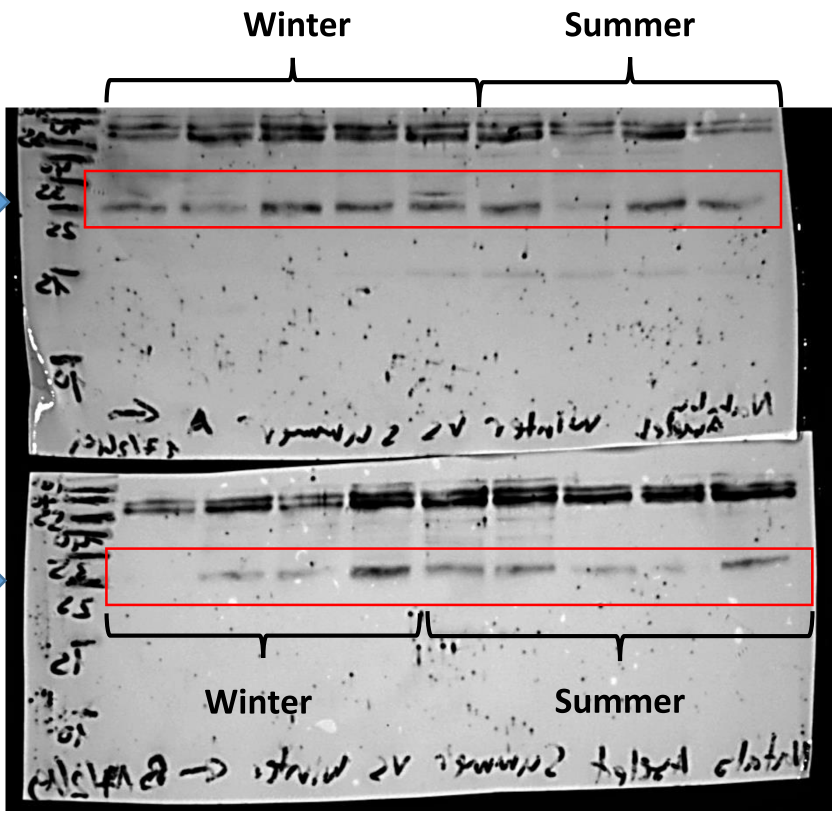

The band at 50 kda could be post-translational modifications and was quantified too but not significant

FAAH protein winter vs. summer (Fig 1)

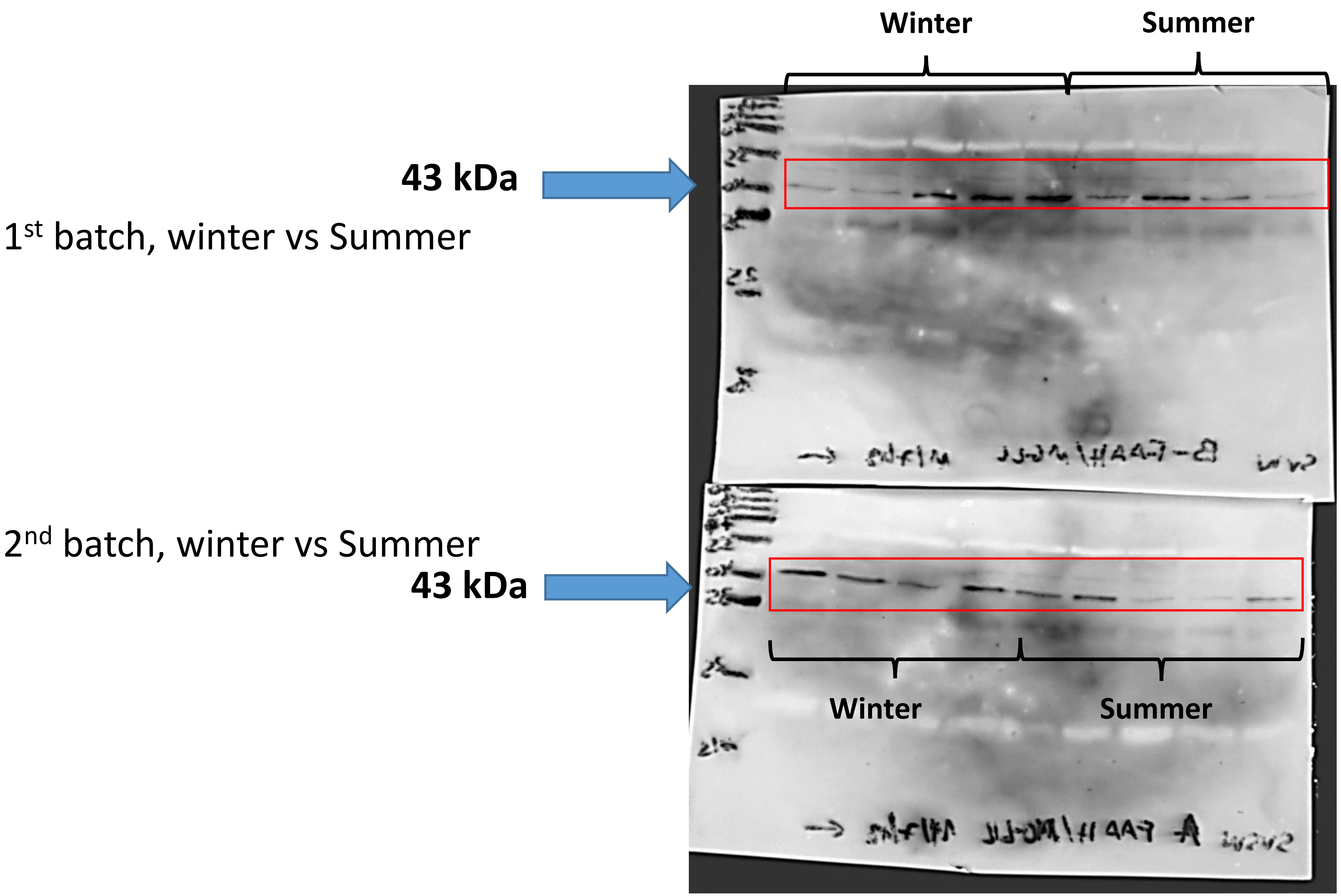

MGLL protein Winter vs. Summer (Fig 1)

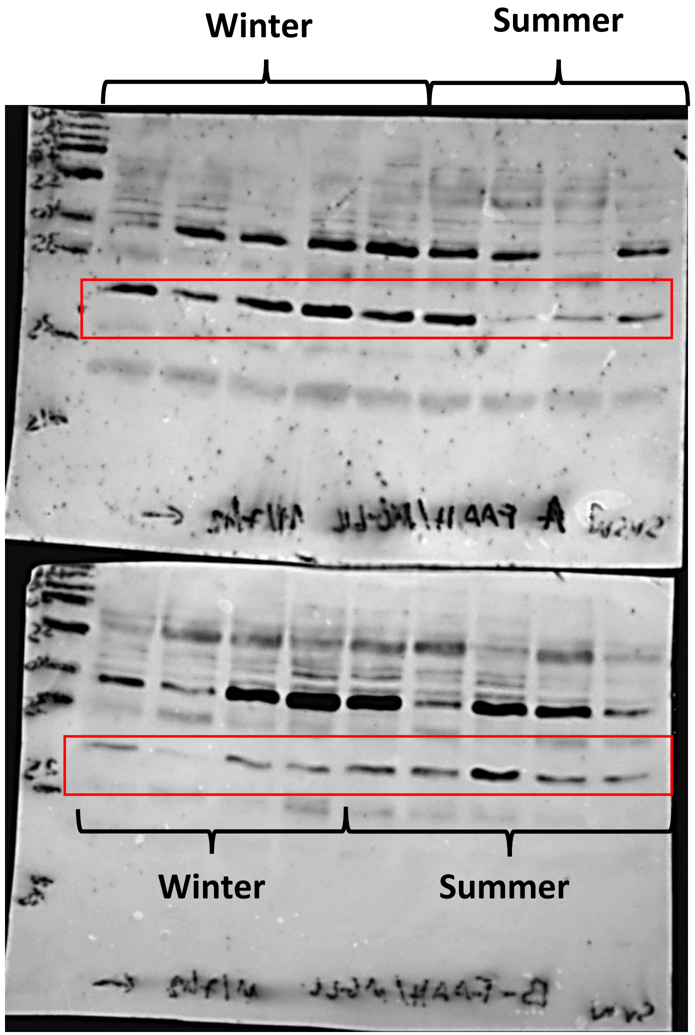

1st batch, winter vs Summer

35 kDa

2nd batch, winter vs Summer

35 kDa

PPARa protein winter vs. summer (Fig 1)

52 kDa

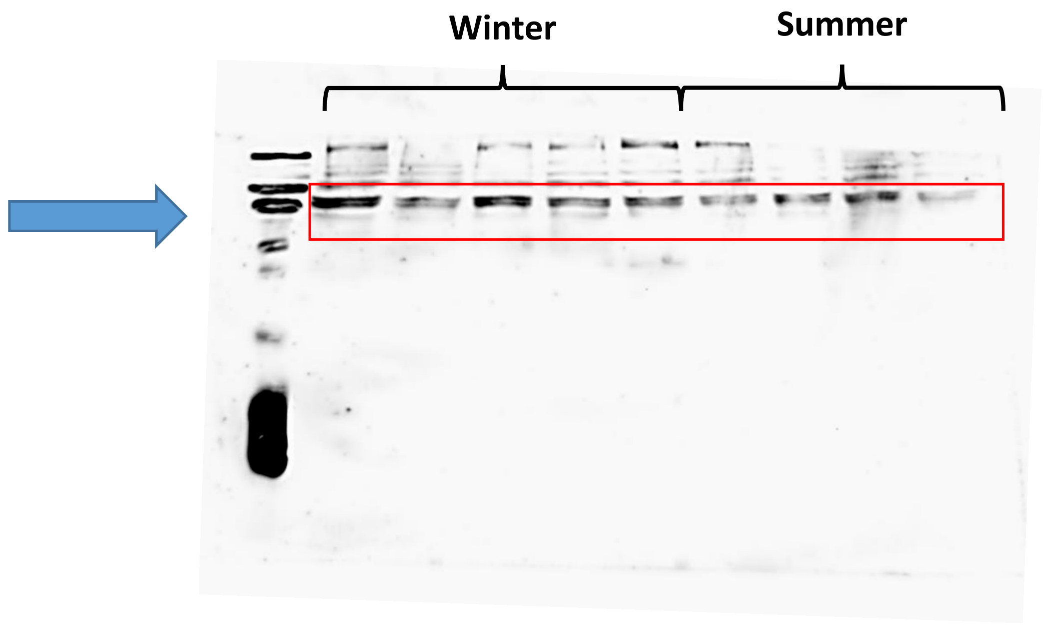

1st batch, winter vs Summer

52 kDa

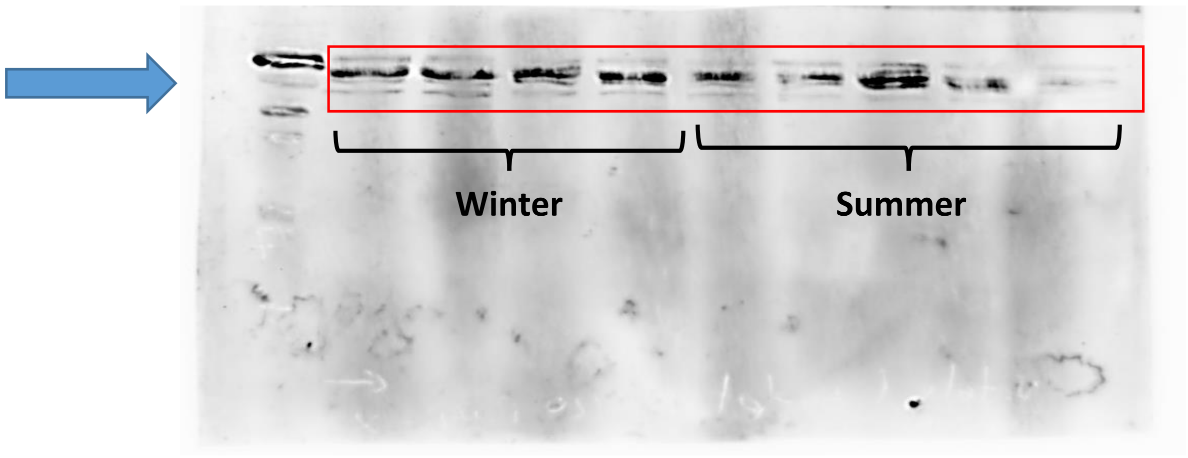

2nd batch, winter vs Summer

TNFa protein winter vs summer (Fig 2)

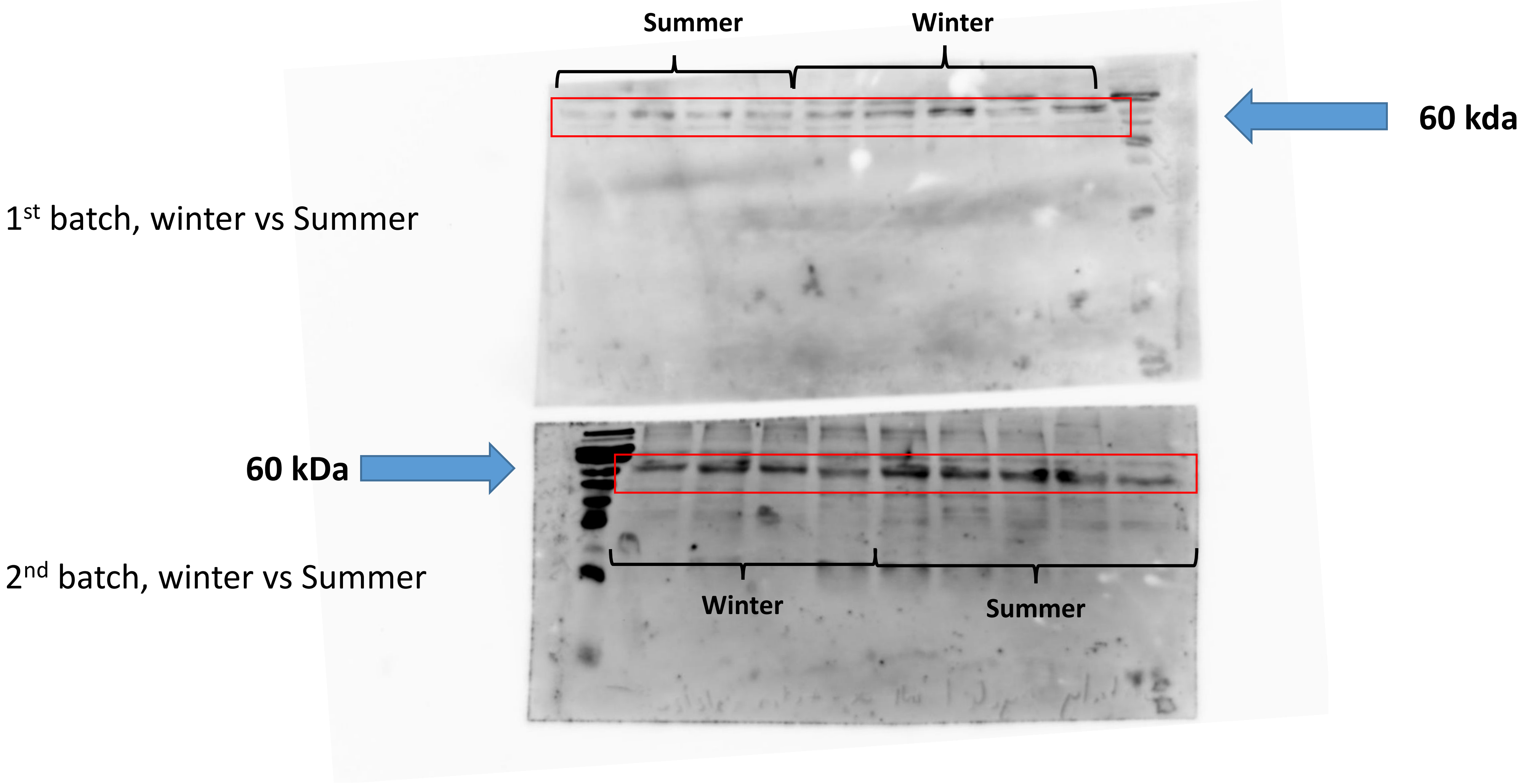

TRPV1 protein winter vs summer (Fig 2)

38 kDa

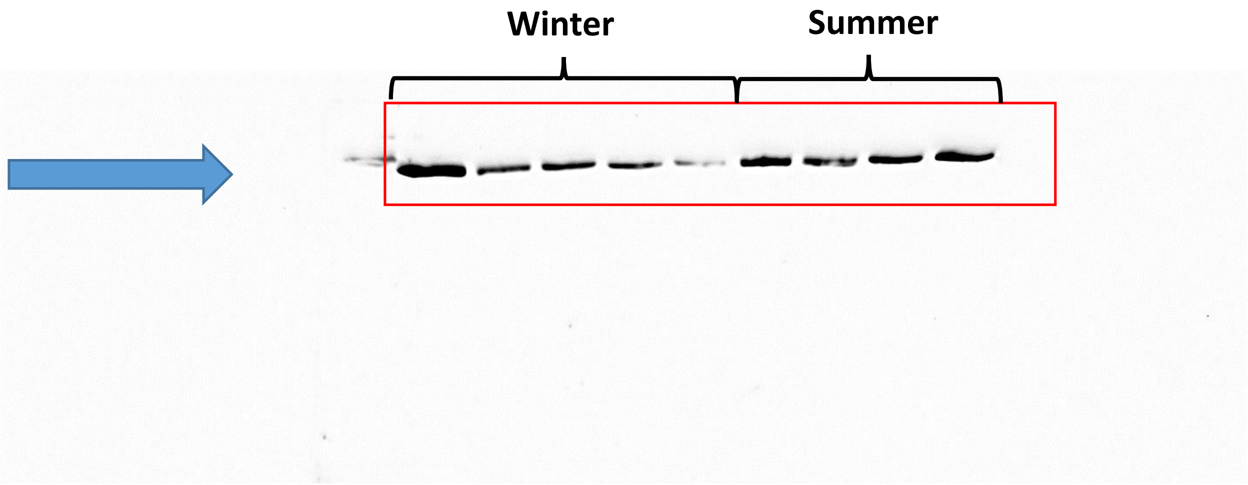

1st batch, winter vs Summer

38 kDa

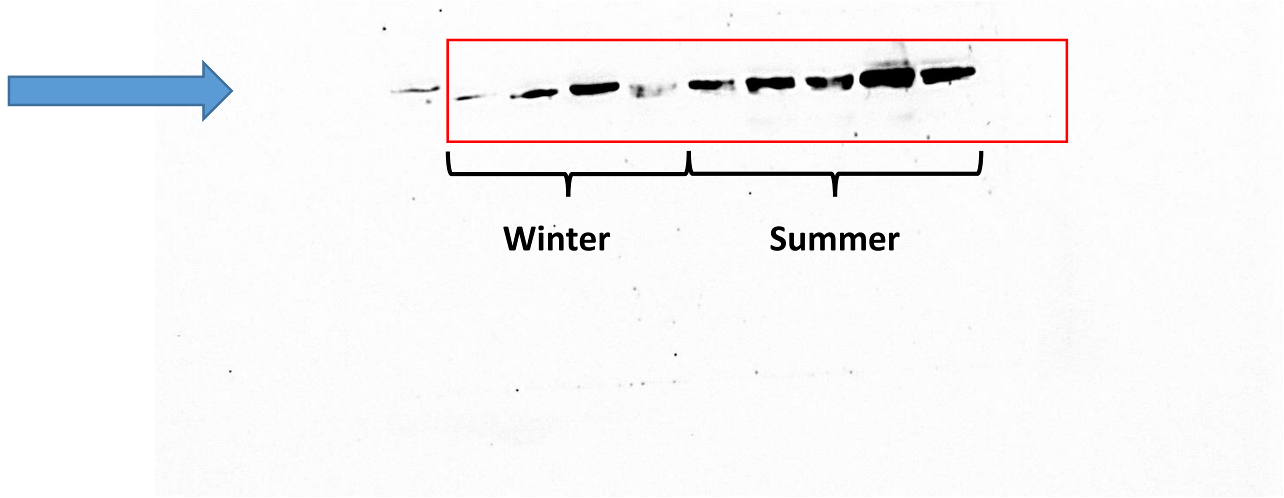

2nd batch, winter vs Summer

Actin protein- number 1 winter vs summer (fig 1)

45 kDa-shown in Fig 1

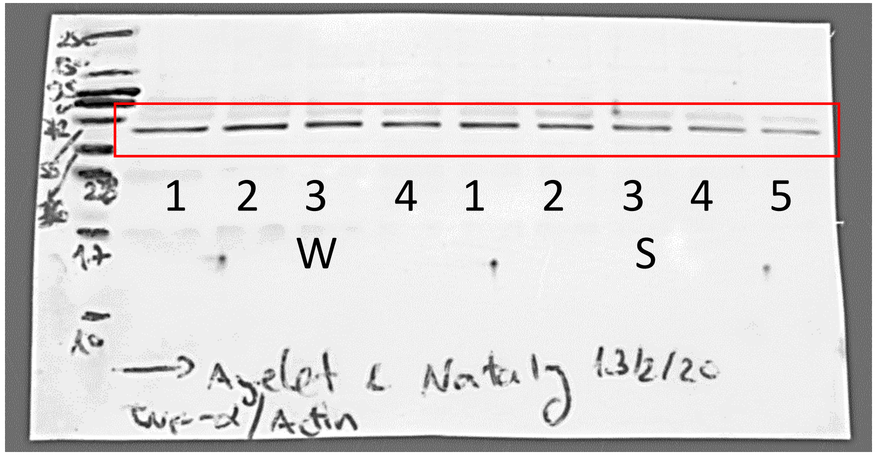

Actin protein- number 2 winter vs. summer (shown in fig 2)

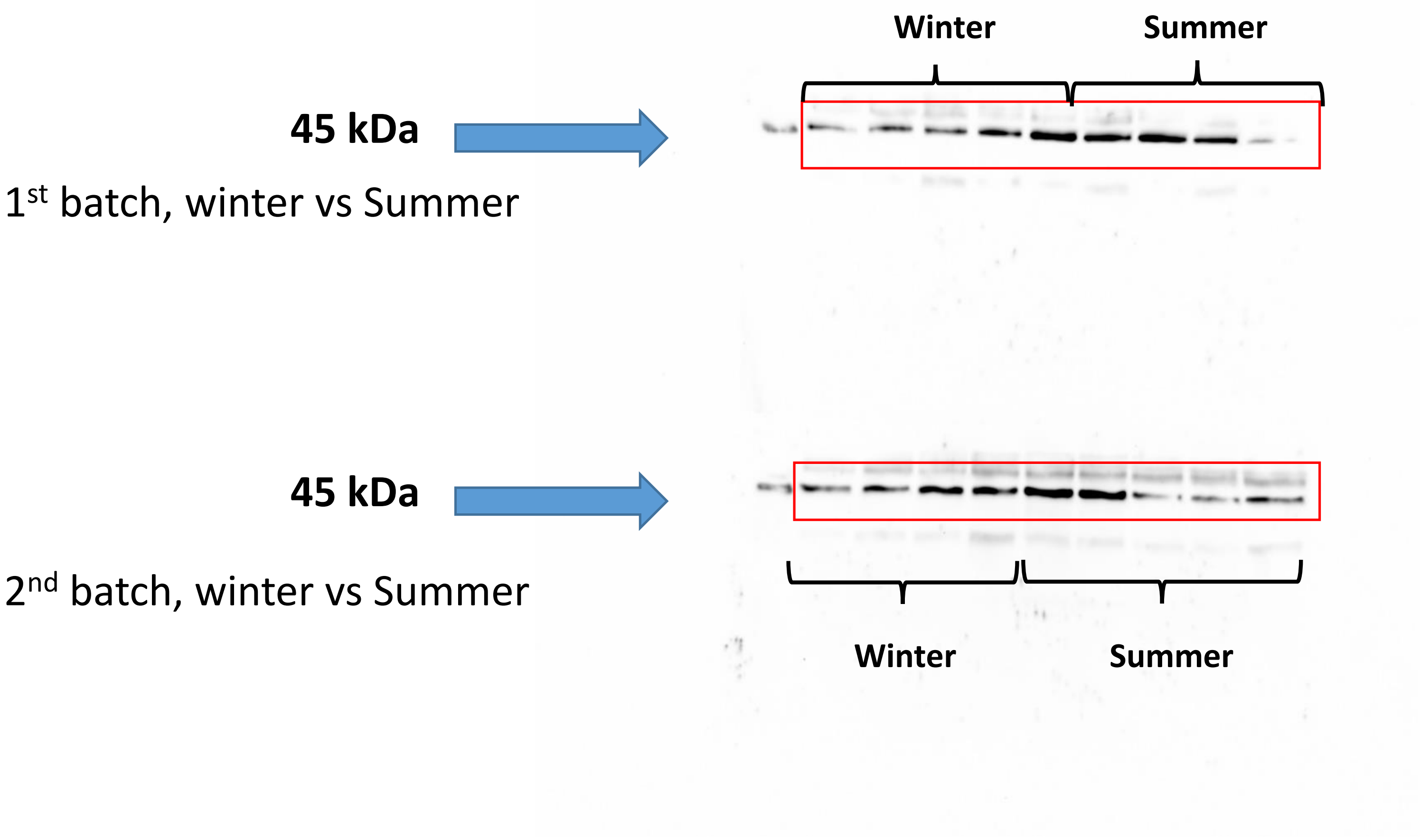

Actin protein- number 3 winter vs summer

1st batch, winter vs Summer

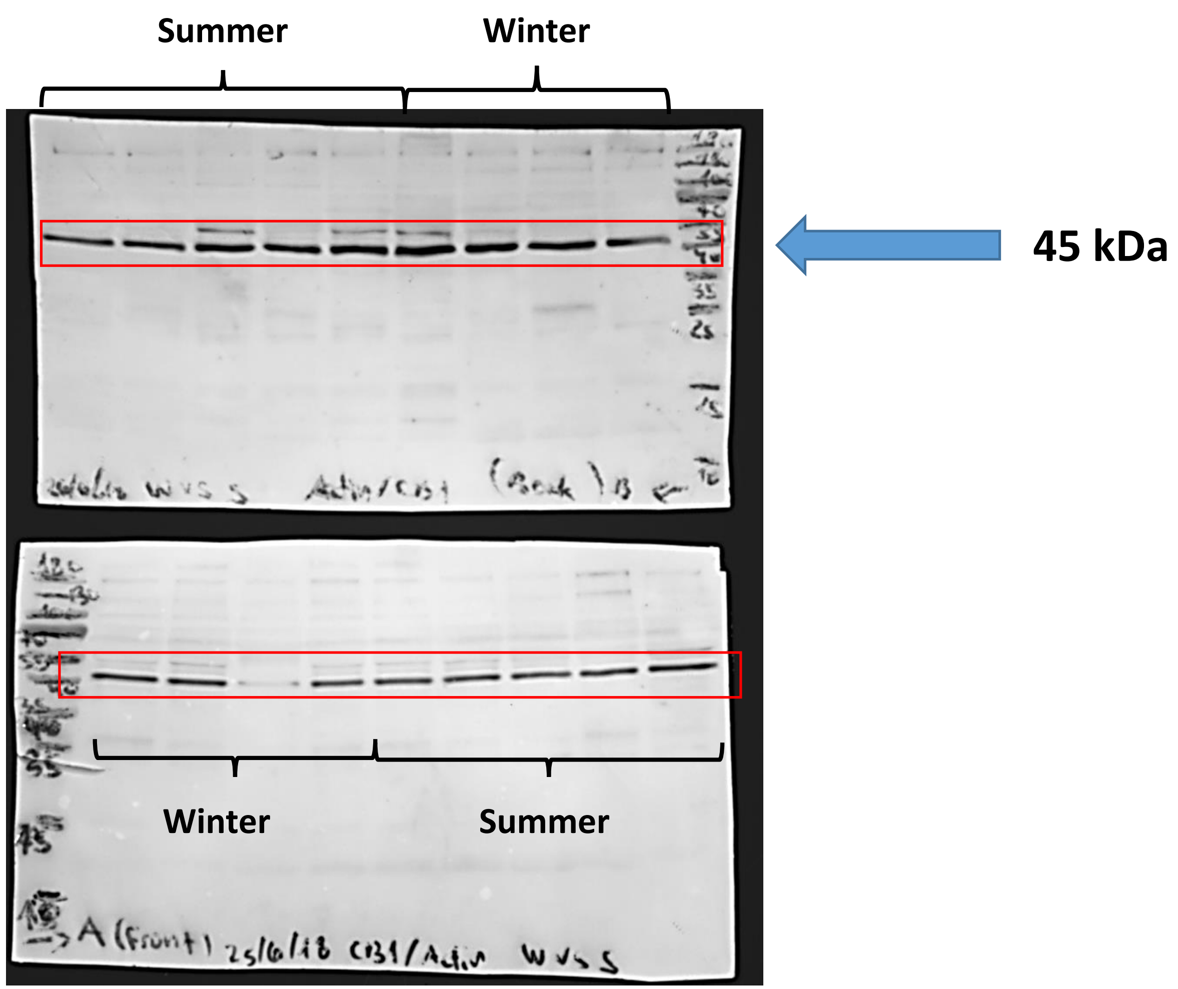

45 kDa

2nd batch, winter vs Summer

CB1 protein in Cooled vs. not cooled (2 runs)- shown in fig 3

60 kDa

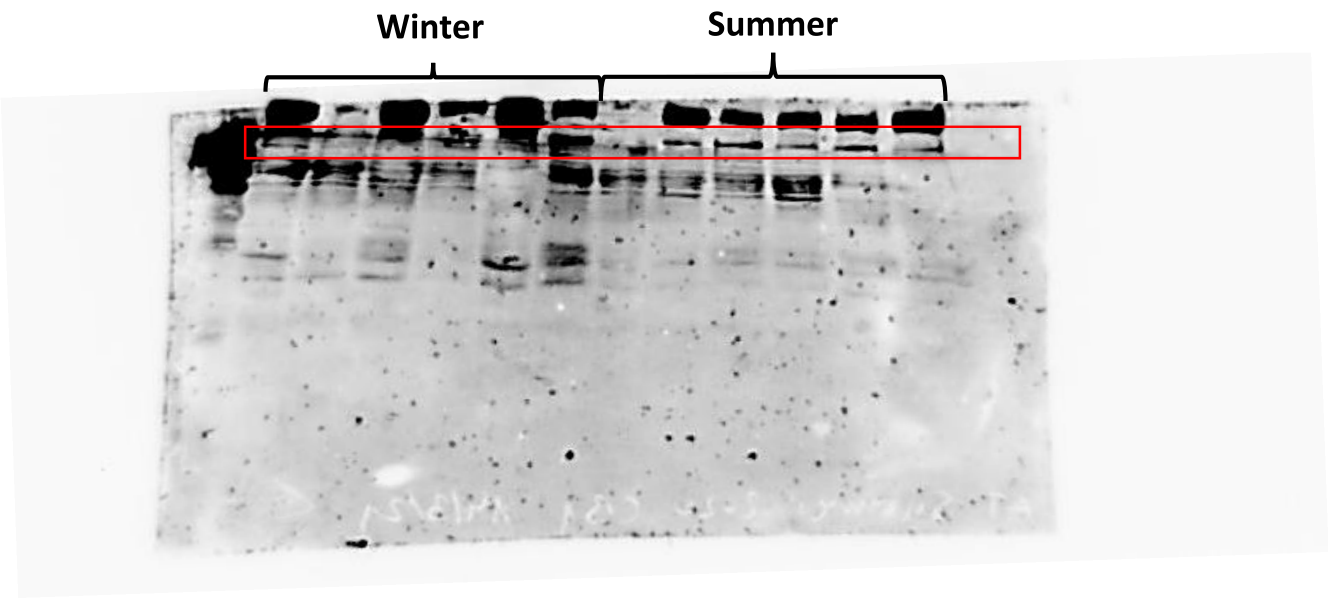

Second run. This was done with another marker in the middle. We presented the above blot even though the background is murky

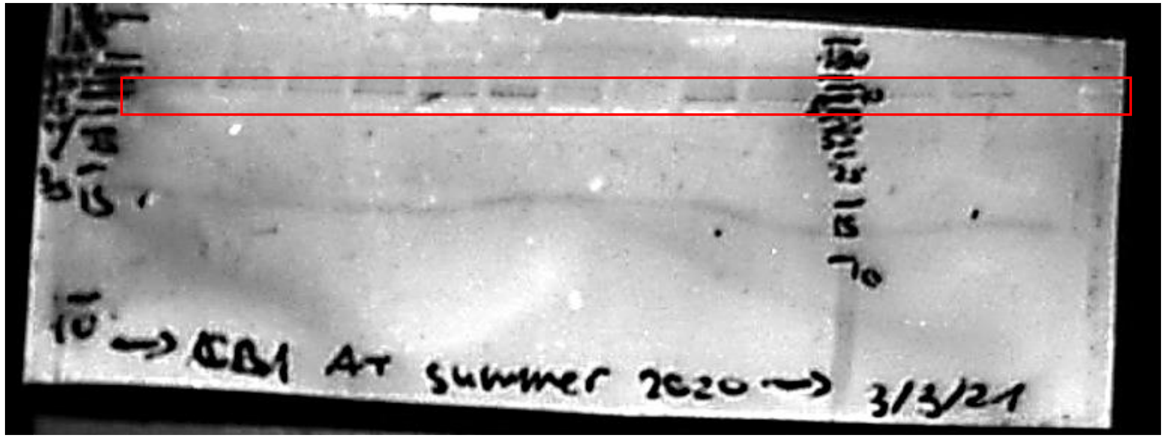

CB2 Cooled vs. not cooled (not shown)

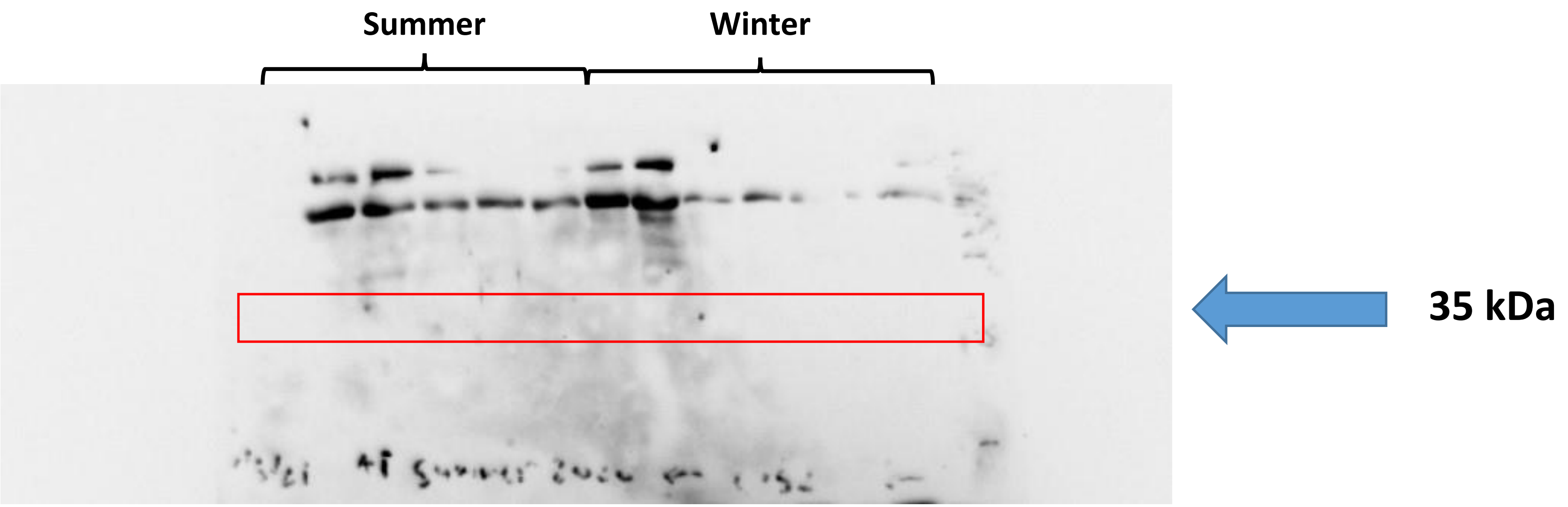

FAAH and MGLL proteins Cooled vs. not cooled (shown in Fig 3)

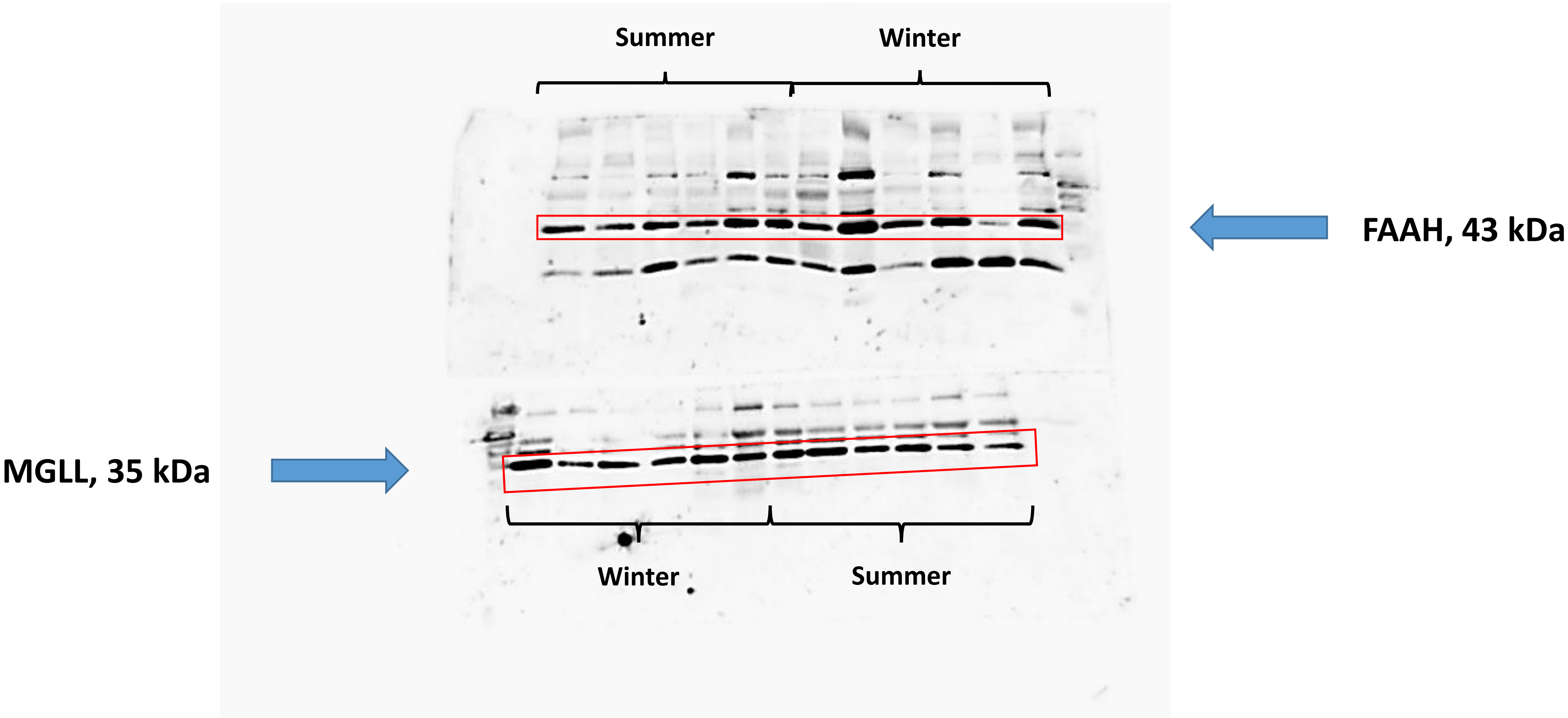

PPARa protein Cooled vs. not cooled (shown in fig 3)

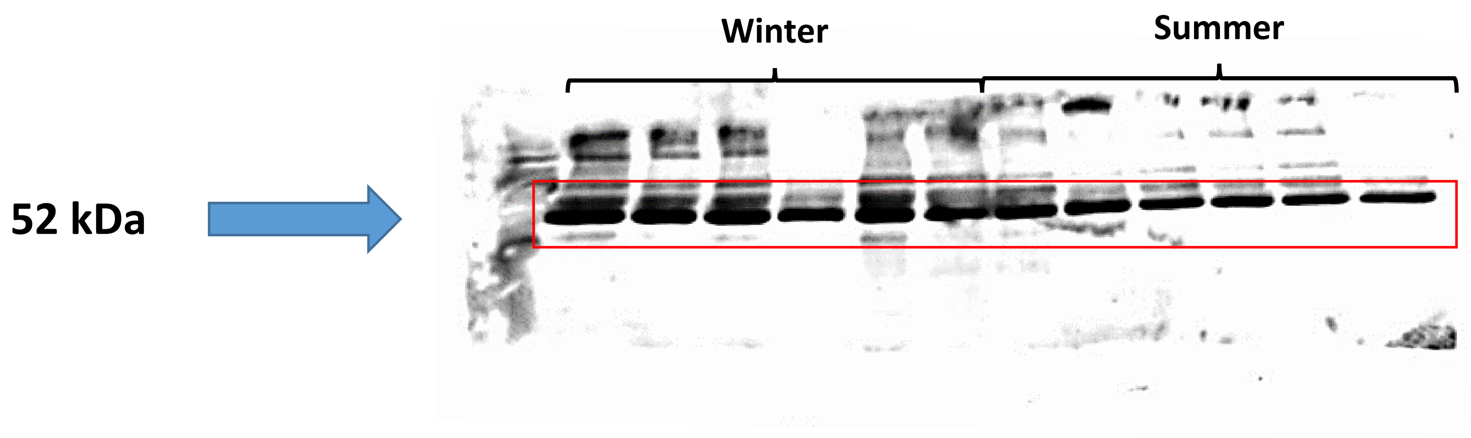

Actin number 2 Cooled vs. not cooled (shown in fig 3)

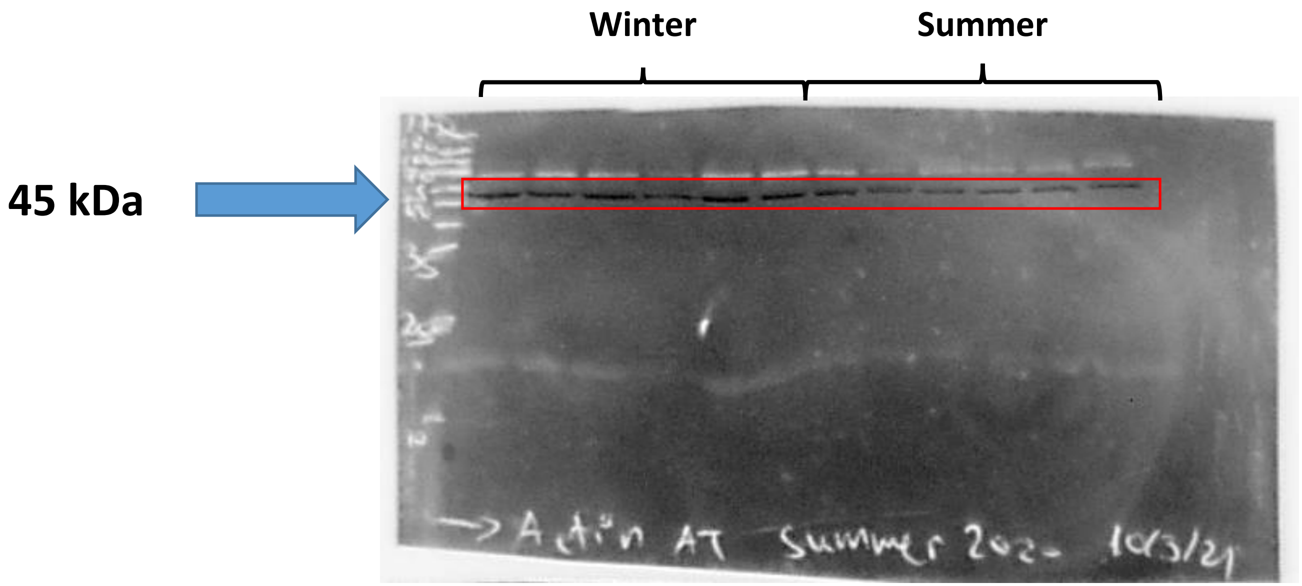

TNFa protein Cooled vs. not cooled (Shown in fig 4)

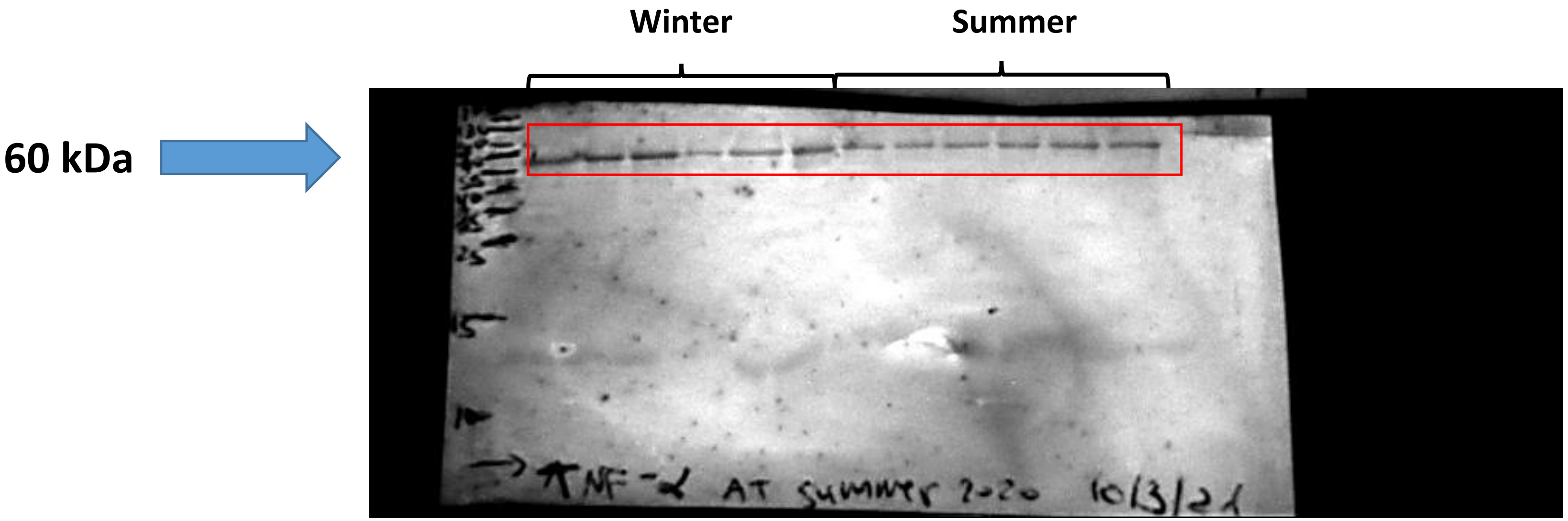

Actin number 1 and TRPV1 proteins Cooled vs. not cooled (shown in Fig 4)

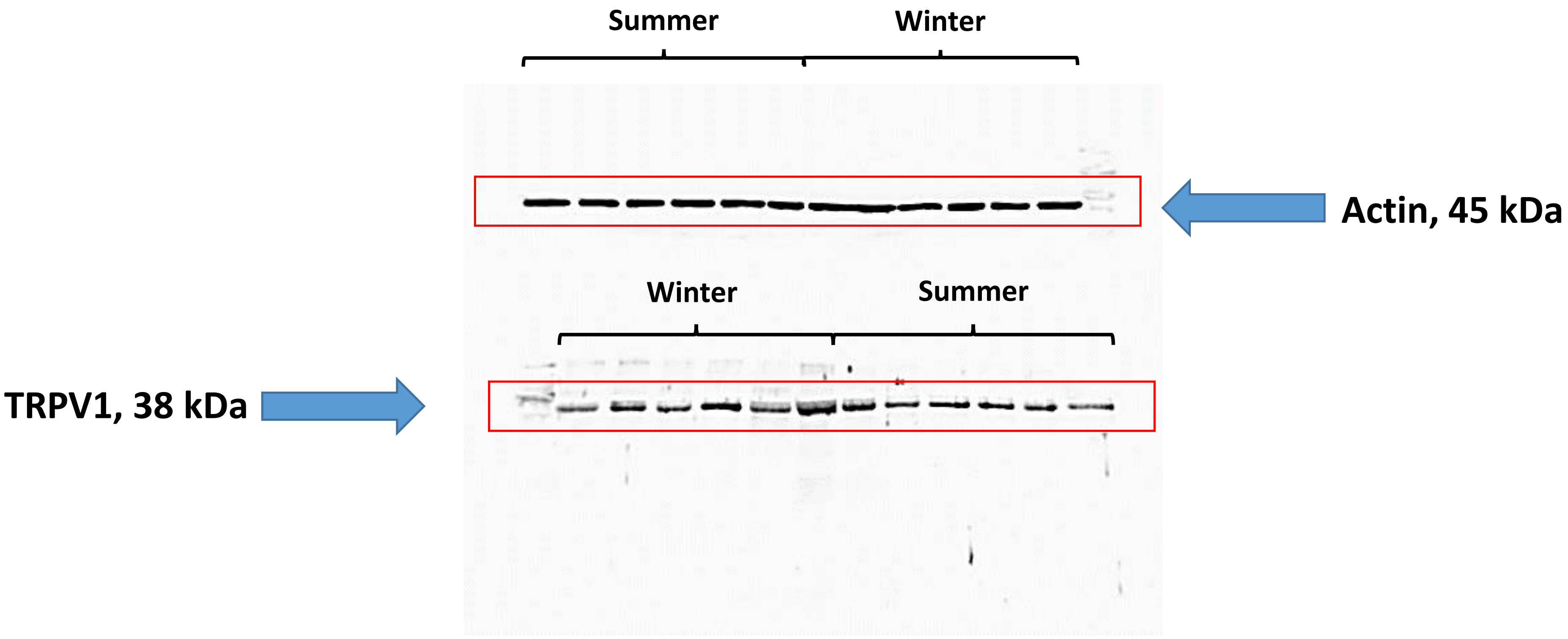

Supplement: Supplementary file 1 [file animals-12-00795-s001.zip › animals-1616207-supplementary.pdf]
